# Matsuda–Heck reaction with arenediazonium tosylates in water

**DOI:** 10.3762/bjoc.11.41

**Published:** 2015-03-16

**Authors:** Ksenia V Kutonova, Marina E Trusova, Andrey V Stankevich, Pavel S Postnikov, Victor D Filimonov

**Affiliations:** 1 Department of Biotechnology and Organic Chemistry, National Research Tomsk Polytechnic University, 634050 Tomsk, Russia; 2 Laboratory of Materials and Technologies of Liquid Crystal Devices, Institute of Chemistry of New Materials NAS Belarus, 220141, Minsk, Belarus; 3 Department of General and Inorganic Chemistry, National Research Tomsk Polytechnic University, 634050 Tomsk, Russia

**Keywords:** alkyl cinnamates, diazonium salts, Matsuda–Heck reaction, microwave-assisted synthesis, stilbenes

## Abstract

An environmentally friendly Matsuda–Heck reaction with arenediazonium tosylates has been developed for the first time. A range of alkenes was arylated in good to quantitative yields in water. The reaction is significantly accelerated when carried out under microwave heating. The arylation of haloalkylacrylates with diazonium salts has been implemented for the first time.

## Introduction

Diazonium salts are known as one of the most valuable building blocks in organic synthesis [1]. Today, their most common use is the construction of carbon–carbon bonds in palladium-catalyzed reactions. Starting with the work of Matsuda’s group [2], who used a diazonium salt as a high-reactive electrophile for a Heck reaction, the Matsuda–Heck reaction does require the addition of neither bases nor ligands and is carried out under very mild conditions [3]. Furthermore, diazonium salts are more often prepared from commercially available anilines than from haloarenes or triflates.

Typically, alcohols, THF or DMF are used as solvents for Matsuda–Heck reactions. One of the most rapidly developing trends in organic synthesis is the carrying out of reactions in water following a «Green chemistry» approach [4]. In 2012, examples of the Matsuda–Heck arylation of styrene and acrylic acid esters with arenediazonium tetrafluoroborates in water and catalyzed with in situ formed Pd nanoparticles [5] or agarose-supported Pd nanoparticles [6] have been reported. Superparamagnetic Pd–ZnFe_2_O_4_ MNPs have been shown to be effective catalysts for the Matsuda–Heck arylation of styrene and ethyl acrylate in water [7]. It is noteworthy that these catalysts are not commercially available and must be synthesized from Pd(OAc)_2_. Roglands et al. prepared a range of *tert*-butyl cinnamates and stilbenes from arenediazonium tetrafluoroborates in water with 5 mol % of commercial PdCl_2_(CH_3_CN)_2_ as a catalyst. The yields of cinnamates were rather high, while stilbenes were obtained with modest yields [8]. Up to date only one example of a simple palladium diacetate-catalyzed arylation of alkenes by diazonium salts in water has been described by the group of Vallribera [9]. Arenediazonium tetrafluoroborates bearing only electron-donating groups (with the exception of chlorine) were effectively coupled with ethyl and *tert*-butyl acrylates only, while ethyl vinyl ketone, styrenes, allyl acetate, allyl benzene and methyl vinyl carbinol were rather less reactive under these conditions, and products were obtained with low to modest yields [9].

Previously, we described a novel type of diazonium salt – arenediazonium tosylates (ADT), which differ from arenediazonium tetrafluoroborates in their higher stability at a dry state and their good solubility in water [10]. It was also shown that ADT are more reactive than arenediazonium tetrafluoroborates in a iodo- [11] and an azidodediazoniation reaction [12]. Another ADT advantage is their safety against explosion accidents, because their decomposition energy is less than 800 J/g [10,12]. In this work, we present a fast, environmentally friendly, low palladium loading method of the Matsuda–Heck arylation of the different alkenes with ADT in water under microwave irradiation.

## Results and Discussion

**Scheme 1:**
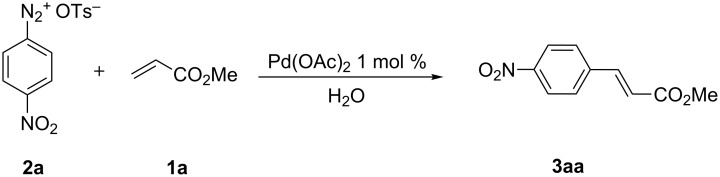
Arylation of methyl acrylate (**1a**) with arenediazonium tosylate **2a**.

Our preliminary study was dedicated to the optimization of the alkene arylation conditions. Methyl acrylate (**1a**) was chosen as a model substrate as it is highly reactive in standard Matsuda–Heck reactions. Therefore, a solution of 4-nitrobenzenediazonium tosylate (**2a**) in water was allowed to react with 1.2 equiv of methyl acrylate (**1a**) in the presence of 1 mol % Pd(OAc)_2_ at rt, at 75 °C with standard heating and at 75 °C based on a microwave-enhanced protocol (Scheme 1, Table 1).

**Table 1:** Arylation of methyl acrylate (**1a**) with arenediazonium tosylate **2a**. Study of reaction conditions.

Entry	*T* (°C)	*t* (min)	Yield of **3aa** (%)

1	rt	80	96
2	75	20	94
3^a^	75	1	97

^a^Reaction was performed in a microwave reactor.

The reaction was carried out at room temperature and methyl 4-nitrocinnamate (**3aa**) was obtained in 96% yield after 80 min (Table 1, entry 1). The increased stability of ADT in organic solvents allowed an increase of the reaction temperature to 75 °C (Table 1, entry 2) which gave the desired compound in comparable yield in 20 min.

It is well-known that microwave irradiation can significantly reduce the reaction time for a wide range of transformations, including Heck reactions, when compared with standard heating [13]. Thus, the aim of our next experiment was the reduction of the reaction time by using microwave irradiation (Table 1, entry 3). By means of this protocol **3aa** was obtained with an almost quantitative yield with a reaction time of only 1 min. Carrying out the reaction with 1 mol % of Pd(OAc)_2_ in water at 75 °C in a microwave reactor was selected as the best reaction conditions.

**Scheme 2:**
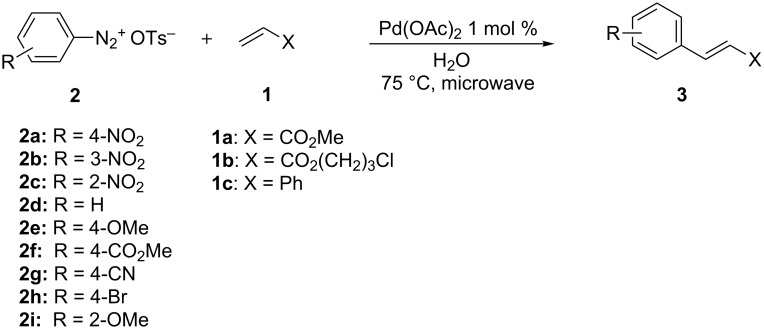
Arylation of alkenes with ADT.

To evaluate the scope of this reaction, a variety of arenediazonium tosylates **2a–i** and a range of alkenes **1a–c** were used (Scheme 2, Table 2).

**Table 2:** Arylation of alkenes with arenediazonium tosylates in water under microwave heating.

Entry	**1**	**2**	*T* (min)	Yield of **3** (%)^a^

1	**1a**	**2a**	1	97
2	**1a**	**2b**	1	92
3	**1a**	**2c**	1	92
4	**1a**	**2d**	1	86
5	**1a**	**2d**	120 (16 h [9])	84^b^ (88 [9])
6	**1a**	**2e**	2	96
7	**1a**	**2f**	1	92
8	**1a**	**2g**	2	94
9	**1a**	**2h**	5	88^c^
10	**1a**	**2i**	6	93^c^
11	**1b**	**2a**	1	90
12	**1b**	**2b**	2	72
13	**1b**	**2g**	3	69
14	**1c**	**2a**	10	67
15	**1c**	**2a**	14 h	42^b^
16	**1c**	**2b**	10	52
17	**1c**	**2e**	50	50
18	**1c**	**2f**	15	65

^a^Reaction conditions: ADT (1 mmol), alkene (1.2 mmol), Pd(OAc)_2_ 1 mol %; isolated yield for pure products; ^b^reaction was performed under rt; ^c^Pd(OAc)_2_ 2.5 mol % were used.

Our method displays a general applicability. The arylation of methyl acrylate (**1a**) was efficient with ADT, substituted by both electron-withdrawing (Table 2, entries 1–3, 7–9) and electron-donating (Table 2, entries 4–6, 9) groups and methyl cinnamates were prepared with good to excellent yields and with excellent purity. Two exceptions are the reaction of ADT **2h** (Table 2, entry 9) and **2i** (Table 2, entry 10), which proofed to be less reactive, so that the complete conversion was only achieved with 2.5 mol % of palladium diacetate. No side-products were detected including phenols (as monitored by GC–MS and HPLC). The latter are often formed if diazonium salts take part in reactions in water and under heating. It is noteworthy that solid methyl cinnamates **3aa–3ac**, **3ae–3ah** can be filtered and purified by simply washing them with water and do not need an additional purification in the absence of organic solvents.

The reaction of ADT with 3-chloropropyl acrylate (**1b**) allows the obtainment of valuable 3-chloropropyl cinnamates (Table 2, entries 11–13) with good to excellent yields. Haloalkyl cinnamates are important building blocks in the synthesis of nitric oxide-donor hybrid drugs for the treatment of cardiovascular disease and cancer [14–17]. Previously, haloalkyl cinnamates were synthesized by the *O*-alkylation of cinnamic acids with dihaloalkanes or haloalkyl alcohols [14,16,18] or by the ring opening of tetrahydrofuran with cinnamic acid anhydrides or haloanhydrides, catalyzed by yttrium trichloride [19], titanium chloride, stannic chloride or allylsamarium bromide [20] under harsh conditions with modest yields. The synthesized 3-chloropropyl cinnamates **3ba, 3bb, 3bg** were not reported before.

Styrene (**1c**) is less reactive than acrylates in Heck-type reactions. Notwithstanding, a range of stilbenes was synthesized stereoselectively with good yields and purity (Table 2, entries 14–18).

## Conclusion

In summary, a fast, simple, eco-friendly Heck arylation protocol in water with stable, safe and water-soluble arenediazonium tosylates is described. It was shown that in most cases the microwave irradiation significantly decreases the reaction time to several minutes. High yields for electron-deficient alkenes and moderate yields for electron-rich alkenes were obtained. The reaction has a broad range and gives the desired products with a high purity.

## Experimental

All starting materials were ACS grade and were employed without further purification. Arenediazonium tosylates **2** were prepared by a previously described method [10]. HPLC analysis was conducted with an Agilent 1200 instrument fitted with an Eclipse Plus C18 column (5 µm, 4.6 × 150 mm) and a UV detector. GC–MS (EI) measurements were obtained with an Agilent 7890/5975C instrument. ^1^H, ^13^C NMR and IR spectra were recorded on a Bruker Avance 300 and a Perkin Elmer BXII, respectively. Melting points were obtained with a melting point system MP50, Mettler Toledo (values are given uncorrected). HPLC–MS measurements were obtained with a Thermo Scientific DFS High Resolution GC–MS. Microwave heating was carried out at 2455 MHz with a CEM Discover System (model 908010) from MATTHEWS, NC (USA).

### Arylation of alkenes **1** with ADT **2** under microwave irradiation: general procedure

To the solution of ADT (1 mmol) in H_2_O (10 mL) at room temperature were added alkene (1.2 mmol) and Pd(OAc)_2_ (0.010 mmol, 2.3 mg). The reaction mixture was heated to 75 °C under stirring by using a microwave reactor in open-vessel mode with a power of 50 W. The conversion of ADT was monitored every minute with a diazonium test with 2-naphthol. When the diazonium test was negative, the reaction mixture was cooled and in the case of solid methyl cinnamates **3aa**–**3ac** and **3ae**–**3ah** the product was filtered, washed with water (25 mL), and dried on air. Otherwise the reaction mixture was extracted twice with dichloromethane (10 mL), the organic layer was filtered through a pad of silica and dried over anhydrous Na_2_SO_4_. The solvent was removed in a rotary evaporator under reduced pressure and the crude product was purified by flash chromatography.

**3-Chloropropyl (*E*)-3-(4-nitrophenyl)acrylate (3ba):** Yield 90%, 0.90 mmol, pale yellow solid, mp 86 °C; ^1^H NMR (300 MHz, DMSO-*d*_6_) δ 8.23 (d, *J* = 8.7 Hz, 2H), 8.00 (d, *J* = 8.7 Hz, 2H), 7.77 (d, *J* = 16.2 Hz, 1H), 6.84 (d, *J* = 16.2 Hz, 1H), 4.28 (t, 2H), 3.76 (t, 2H), 2.11 (m, 2H); ^13^C NMR (75 MHz, DMSO-*d*_6_) δ 165.6, 148.0, 142.4, 140.4, 129.5, 123.9, 122.2, 61.4, 41.9, 31.1; EI–MS *m*/*z*: 269 [M]^+^; HRMS *m*/*z*: calcd for C_12_H_12_NO_4_Cl, 269.0449; found, 269.0452.

**3-Chloropropyl (*E*)-3-(3-nitrophenyl)acrylate (3bb):** Yield 72%, 0.72 mmol, pale yellow solid, mp 75 °C; ^1^H NMR (300 MHz, DMSO-*d*_6_) δ 8.54 (s, 1H), 8.24–8.17 (m, 2H), 7.80 (d, *J* = 16.2 Hz, 1H), 7.69 (m, 1H), 6.84 (d, *J* = 16.2 Hz, 1H), 4.27 (t, 2H), 3.77 (t, 2H), 2.11 (m, 2H); ^13^C NMR (75 MHz, DMSO-*d*_6_) δ 165.7, 148.2, 142.2, 135.9, 134.1, 130.4, 124.6, 123.0, 120.9, 61.3, 41.9, 31.2; HRMS *m*/*z*: calcd. for C_12_H_12_NO_4_Cl, 269.0449; found, 269.0456.

**3-Chloropropyl (*E*)-3-(4-cyanophenyl)acrylate (3bg):** Yield 69%, 0.69 mmol, white solid, mp 58 °C; ^1^H NMR (300 MHz, DMSO-*d*_6_) δ 7.93–7.85 (m, 4H), 7.74 (d, *J* = 16.2 Hz, 1H), 6.80 (d, *J* = 16.2 Hz, 1H), 4.27 (t, 2H), 3.75 (t, 2H), 2.11 (m, 2H); ^13^C NMR (75 MHz, DMSO-*d*_6_) δ 165.6, 142.6, 138.5, 132.7, 129.0, 121.4, 118.5, 112.3, 61.3, 41.8, 31.1; HRMS *m*/*z*: calcd. for C_13_H_12_NO_2_Cl, 249.0551; found, 249.0548.

## Supporting Information

File 1 Additional experimental data.
