# Supplementary material for: Matsuda–Heck reaction with arenediazonium tosylates in water
Source: Beilstein J Org Chem. 2015 Mar 16;11:358–62. doi: 10.3762/bjoc.11.41 (PMC4419561; doi:10.3762/bjoc.11.41)
Supplement: File 1 — Additional experimental data. [file Beilstein_J_Org_Chem-11-358-s001.pdf]

Supporting Information

for

Matsuda–Heck reaction with arenediazonium tosylates in water

Ksenia V. Kutonova[1], Marina E. Trusova[1], Andrey V. Stankevich[2], Pavel S. Postnikov[3*,§] and Victor D. Filimonov[1]

Address: [1]Department of Biotechnology and Organic Chemistry, National Research Tomsk Polytechnic University, 634050 Tomsk, Russia, [2]Laboratory of Materials and Technologies of Liquid Crystal Devices, Institute of Chemistry of New Materials NAS Belarus, 220141, Minsk, Belarus and [3]Department of General and Inorganic Chemistry, National Research Tomsk Polytechnic University, 634050 Tomsk, Russia

*Corresponding author

Email: Pavel S. Postnikov - postnikov@tpu.ru

[§]Fax +7 (3822)563637

Additional experimental data

1. GENERAL INFORMATION

All starting materials were ACS grade and were employed without further purification. Arenediazonium tosylates **2** were prepared by a previously described method [1]. HPLC analysis was conducted with an Agilent 1200 instrument fitted with an Eclipse Plus C18 column (5 µm, 4.6 × 150 mm) and UV detector. GC–MS (EI) measurements were obtained with an Agilent 7890/5975C instrument. ^{1}H, ^{13}C NMR and IR spectra were recorded on a Bruker Avance 300 and Perkin Elmer BXII instruments, respectively. Melting points were obtained with a melting point system MP50, Mettler Toledo (values are given uncorrected). HPLC–MS measurements were obtained with a Thermo Scientific DFS High Resolution GC–MS. Microwave heating was carried out with a CEM Discover System (model 908010) at 2455 MHz from MATTHEWS, NC (USA).

2. EXPERIMENTAL PROCEDURE

To the solution of ADT (1 mmol) in H_2O (10 mL) at room temperature were added alkene (1.2 mmol) and $Pd(OAc)_2$ (0.010 mmol; 2.3 mg). The reaction mixture was heated and held at 75 °C with stirring using a microwave reactor in open-vessel mode with power 50 W. Conversion of ADT was monitored every minute until a negative diazonium test with 2-naphthol. When the diazonium test was negative, the reaction mixture was cooled and in the case of solid methyl cinnamates 3aa–3ac and 3ae–3ah the product was filtered, washed with water (25 mL), and dried on air. Otherwise the reaction mixture was extracted twice with dichloromethane (10 mL), the organic layer was filtered through a pad of silica and dried over anhydrous Na_2SO_4. The solvent was removed in a rotary evaporator under reduced pressure and the crude product was purified by flash chromatography.

3. CHARACTERIZATION FOR PRODUCTS

Methyl (*E*)-3-(4-nitrophenyl)acrylate (**3aa**): Yield 97%, 0.97 mmol, pale yellow solid, mp 138 °C. Analytical data are identical to the reported data [2]. ^{1}H NMR (300 MHz, $CDCl_3$) δ: 8.24 (d, 2H, J = 8.4 Hz), 7.74-7.65 (m, 3H), 6.56 (d, 1H, J = 16.2 Hz), 3.83 (s, 3H); ^{13}C NMR (75 MHz, $CDCl_3$) δ: 166.5, 148.4, 141.9, 140.4, 128.7, 124.2, 122.0, 52.0; MS (EI): m/z= 207 ($[M]^+$).

Methyl (*E*)-3-(3-nitrophenyl)acrylate (**3ab**): Yield 92%, 0.92 mmol, pale yellow solid, mp 124 °C. Analytical data are identical to the reported data [2]. ^{1}H NMR (300 MHz, $CDCl_3$) δ: 8.34 (s, 1H), 8.20 (d, 1H, J = 8.1 Hz), 7.80 (d, 1H, J = 8.1 Hz), 7.69 (d, 1H, J = 15.9 Hz), 7.57 (m, 1H), 6.53 (d, 1H, J = 15.9 Hz), 3.80 (s, 3H); ^{13}C NMR (75 MHz, $CDCl_3$) δ: 166.2, 148.3, 141.6, 135.7, 133.4, 129.8, 124.3, 122.1, 120.6, 51.8; MS (EI): m/z= 207 ($[M]^+$).

Methyl (*E*)-3-(2-nitrophenyl)acrylate (**3ac**): Yield 92%, 0.92 mmol, pale yellow solid, mp 72 °C. Analytical data are identical to the reported data [2]. ^{1}H NMR (300 MHz, $CDCl_3$) δ: 8.09 (d, 1H,

J = 15.6 Hz), 8.02 (d, 1H, J = 8.4 Hz), 7.67-7.60 (m, 2H), 7.56-7.50 (m, 1H), 6.35 (d, 1H, J = 15.6 Hz), 3.80 (s, 3H); ^{13}C NMR (75 MHz, $CDCl_3$) δ: 166.1, 148.1, 140.0, 133.5, 130.3, 129.0, 124.8, 122.7, 51.9; MS (EI): m/z= 207 ($[M]^+$).

Methyl (*E*)-cinnamate (**3ad**): Yield 86%, 0.86 mmol, pale yellow oil. Analytical data are identical to the reported data [2]. ^{1}H NMR (300 MHz, $CDCl_3$) δ: 7.70 (d, 1H, J = 16.2 Hz), 7.53-7.50 (m, 2H), 7.39-7.36 (m, 3H), 6.44 (d, 1H, J = 16.2 Hz), 3.80 (s, 3H); ^{13}C NMR (75 MHz, $CDCl_3$) δ: 167.4, 144.8, 134.3, 130.3, 128.8, 128.0, 117.7, 51.7; MS (EI): m/z= 162 ($[M]^+$).

Methyl (*E*)-3-(4-methoxyphenyl)acrylate (**3ae**): Yield 96%, 0.96 mmol, white solid, mp 90 °C. Analytical data are identical to the reported data [2]. ^{1}H NMR (300 MHz, $CDCl_3$) δ: 7.64 (d, 1H, J = 15.9 Hz), 7.46 (d, 2H, J = 8.4 Hz), 6.88 (d, 2H, J = 8.4 Hz), 6.30 (d, 1H, J = 15.9 Hz), 3.81 (s, 3H), 3.77 (s, 3H); ^{13}C NMR (75 MHz, $CDCl_3$) δ: 167.7, 161.3, 144.5, 129.7, 127.0, 115.2, 114.3, 55.3, 51.6; MS (EI): m/z = 192 ($[M]^+$).

Methyl (*E*)-4-(3-methoxy-3-oxoprop-1-en-1-yl)benzoate (**3af**): Yield 92%, 0.92 mmol, white solid, mp 125 °C. Analytical data are identical to the reported data [3]. ^{1}H NMR (300 MHz, $CDCl_3$) δ: 8.03 (d, 2H, J = 8.4 Hz), 7.70 (d, 1H, J = 16.2 Hz), 7.57 (d, 2H, J = 8.4 Hz), 6.51 (d, 1H, J = 16.2 Hz), 3.92 (s, 3H), 3.81 (s, 3H); ^{13}C NMR (75 MHz, $CDCl_3$) δ: 167.0, 166.5, 143.5, 138.6, 131.4, 130.1, 128.0, 120.2, 52.4, 52.0; MS (EI): m/z= 220 ($[M]^+$).

Methyl (*E*)-3-(4-cyanophenyl)acrylate (**3ag**): Yield 94%, 0.94 mmol, white solid, mp 138 °C. Analytical data are identical to the reported data [4]. ^{1}H NMR (300 MHz, $CDCl_3$) δ: 7.67 (m, 3H), 7.61 (m, 2H), 6.51 (d, 1H, J = 15.9 Hz), 3.81 (s, 3H); ^{13}C NMR (75 MHz, $CDCl_3$) δ: 166.6, 142.4, 138.6, 132.7, 128.4, 121.4, 118.4, 113.4, 52.1; MS (EI): m/z= 187 ($[M]^+$).

Methyl (*E*)-3-(4-bromophenyl)acrylate (**3ah**): Yield 88%, 0.88 mmol, white solid, mp 89 °C. Analytical data are identical to the reported data [2]. ^{1}H NMR (300 MHz, $CDCl_3$) δ: 7.61 (d, 1H, J = 16.2 Hz), 7.50 (d, 2H, J = 8.1 Hz), 7.36 (d, 2H, J = 8.1 Hz), 6.41 (d, 1H, J = 16.2 Hz), 3.80 (s, 3H); ^{13}C NMR (75 MHz, $CDCl_3$) δ: 167.1, 143.4, 133.1, 132.1, 129.4, 124.5, 118.4, 51.8; MS (EI): m/z= 240 ($[M]^+$);

Methyl (*E*)-3-(2-methoxyphenyl)acrylate (**3ai**): Yield 93%, 0.93 mmol, colorless oil. Analytical data are identical to the reported data [5]. ^{1}H NMR (300 MHz, $CDCl_3$) δ: 7.99 (d, 1H, J = 16.2 Hz), 7.47 (d, 1H, J = 8.1 Hz), 7.31 (m, 1H), 6.95-6.86 (m, 2H), 6.97 (m, 1H), 6.51 (d, 1H, J = 16.2 Hz), 3.83 (s, 3H), 3.77 (m, 3H); ^{13}C NMR (75 MHz, $CDCl_3$) δ: 167.6, 158.0, 140.0, 131.3, 128.6, 123.0, 120.4, 117.9, 110.9, 55.2, 51.3; MS (EI): m/z= 192 ([M]+);

3-Chloropropyl (*E*)-3-(4-nitrophenyl)acrylate (**3ba**): Yield 90%, 0.90 mmol, pale yellow solid, mp 86 °C. ^{1}H NMR (300 MHz, DMSO-d_6) δ: 8.23 (d, 2H, J = 8.7 Hz), 8.00 (d, 2H, J = 8.7 Hz), 7.77 (d, 1H, J = 16.2 Hz), 6.84 (d, 1H, J = 16.2 Hz), 4.28 (t, 2H), 3.76 (t, 2H), 2.11 (m, 2H). ^{13}C

NMR (75 MHz, DMSO-d_6) δ: 165.6, 148.0, 142.4, 140.4, 129.5, 123.9, 122.2, 61.4, 41.9, 31.1. MS (EI): m/z= 269 ($[M]^+$); HR-MS m/z: calcd for $C_{12}H_{12}NO_4Cl$, 269.0449; found, 269.0452.

3-Chloropropyl (*E*)-3-(3-nitrophenyl)acrylate (**3bb**): Yield 72%, 0.72 mmol, pale yellow solid, mp 75 °C. ^{1}H NMR (300 MHz, DMSO-d_6) δ: 8.54 (s, 1H), 8.24-8.17 (m, 2H), 7.80 (d, 1H, J = 16.2 Hz), 7.69 (m, 1H), 6.84 (d, 1H, J = 16.2 Hz), 4.27 (t, 2H), 3.77 (t, 2H), 2.11 (m, 2H). ^{13}C NMR (75 MHz, DMSO-d_6) δ: 165.7, 148.2, 142.2, 135.9, 134.1, 130.4, 124.6, 123.0, 120.9, 61.3, 41.9, 31.2; HR-MS m/z: calcd. for $C_{12}H_{12}NO_4Cl$, 269.0449; found, 269.0456.

3-Chloropropyl (*E*)-3-(4-cyanophenyl)acrylate (**3bg**): Yield 69%, 0.69 mmol, white solid, mp 58 °C. ^{1}H NMR (300 MHz, DMSO-d_6) δ: 7.93-7.85 (m, 4H), 7.74 (d, 1H, J = 16.2 Hz), 6.80 (d, 1H, J = 16.2 Hz), 4.27 (t, 2H), 3.75 (t, 2H), 2.11 (m, 2H). ^{13}C NMR (75 MHz, DMSO-d_6) δ: 165.6, 142.6, 138.5, 132.7, 129.0, 121.4, 118.5, 112.3, 61.3, 41.8, 31.1; HR-MS m/z: calcd. for $C_{13}H_{12}NO_2Cl$, 249.0551; found 249.0548.

(*E*)-1-Nitro-4-styrylbenzene (**3ca**): Yield 67%, 0.67 mmol, pale yellow solid, mp 154 °C. Analytical data are identical to the reported data [6]. ^{1}H NMR (300 MHz, DMSO-d_6) δ: 8.21 (d, 2H, J = 7.5 Hz), 7.85 (d, 2H, J = 7.4 Hz), 7.66 (d, 2H, J = 5.7 Hz), 7.52 (d, 1H, J = 16.5 Hz), 7.42 (m, 4H). ^{13}C NMR (75 MHz, DMSO-d_6) δ: 146.1, 143.9, 136.2, 133.2, 128.8, 128.7, 127.3, 127.1, 126.3, 124.0.

(*E*)-1-Nitro-3-styrylbenzene (**3cb**): Yield 52%, 0.52 mmol, pale yellow solid, mp 93 °C. Analytical data are identical to the reported data [7]. ^{1}H NMR (300 MHz, DMSO-d_6) δ: 8.35 (s, 1H), 8.08 (d, 1H, J = 8.1 Hz), 7.78 (d, 1H, J = 8.1 Hz), 7.53 (m, 3H), 7.40 (m, 2H), 7.33 (d, 1H, J = 6.6 Hz), 7.23 (d, 1H, J = 16.5 Hz), 7.11 (d, 1H, J = 16.5 Hz). ^{13}C NMR (75 MHz, DMSO-d_6) δ: 148.5, 139.0, 136.1, 132.2, 131.6, 129.5, 128.7, 128.4, 126.7, 126.0, 121.9, 120.7.

(*E*)-1-methoxy-4-styrylbenzene (**3ce**): Yield 50%, 0.50 mmol, white solid, mp 140 °C. Analytical data are identical to the reported data [6]. ^{1}H NMR (300 MHz, DMSO-d_6) δ: 7.57-7.53 (m, 4H), 7.38-7.33 (m, 2H), 7.26-7.23 (m, 1H), 7.19 (d, 1H, J = 16.5 Hz), 7.08 (d, 1H, J = 16.5 Hz), 6.95 (d, 2H, J = 8.7 Hz), 3.77 (s, 3H). ^{13}C NMR (75 MHz, DMSO-d_6) δ: 158.9, 137.3, 129.6, 128.6, 128.0, 127.8, 127.1, 126.1, 114.1, 55.2.

Methyl (*E*)-4-styrylbenzoate (**3cf**): Yield 65%, 0.65 mmol, white solid, mp 158 °C. Analytical data are identical to the reported data [3]. ^{1}H NMR (300 MHz, DMSO-d_6) δ: 8.03 (d, 2H, J = 7.8 Hz), 7.55 (m, 4H), 7.39 (m, 2H), 7.30 (m, 1H), 7.22 (d, 1H, J = 16.5 Hz), 7.12 (d, 1H, J = 16.5 Hz), 3.93 (s, 3H). ^{13}C NMR (75 MHz, DMSO-d_6) δ: 166.7, 141.6, 136.5, 131.1, 129.9, 128.7, 128.1, 127.4, 126.7, 126.2, 52.0;

4. NMR and MASS SPECTRA COPIES

Methyl (*E*)-3-(4-nitrophenyl)acrylate (**3aa**):

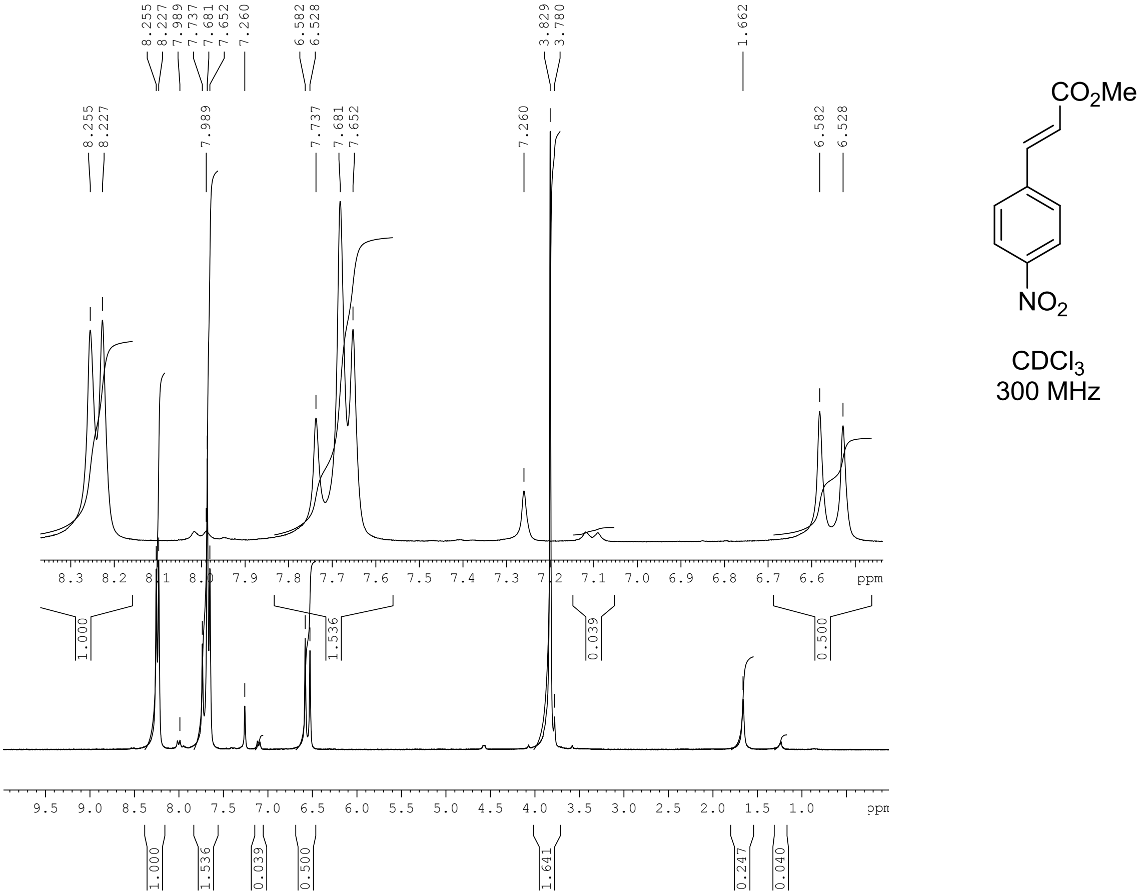

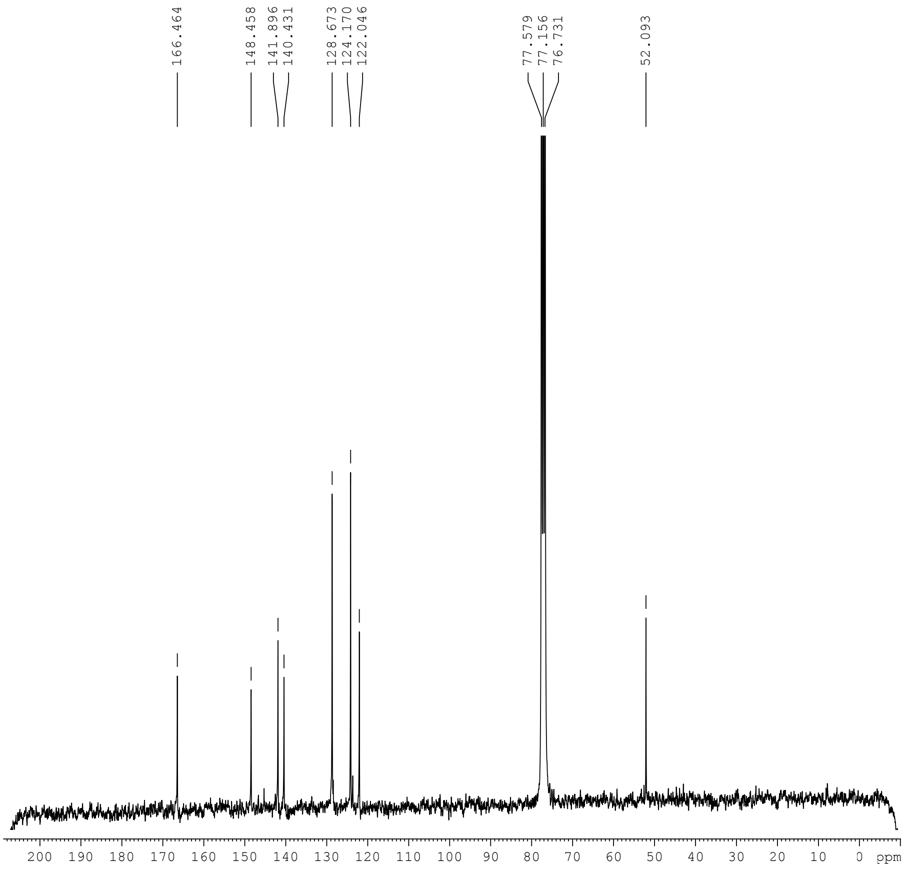
166.464
148.458
141.896
140.431
128.673
124.170
122.046
77.579
77.156
76.731
52.093
200 190 180 170 160 150 140 130 120 110 100 90 80 70 60 50 40 30 20 10 0 ppm

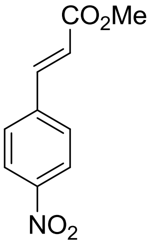
CO2Me
NO2

$CDCl_3$
75 MHz

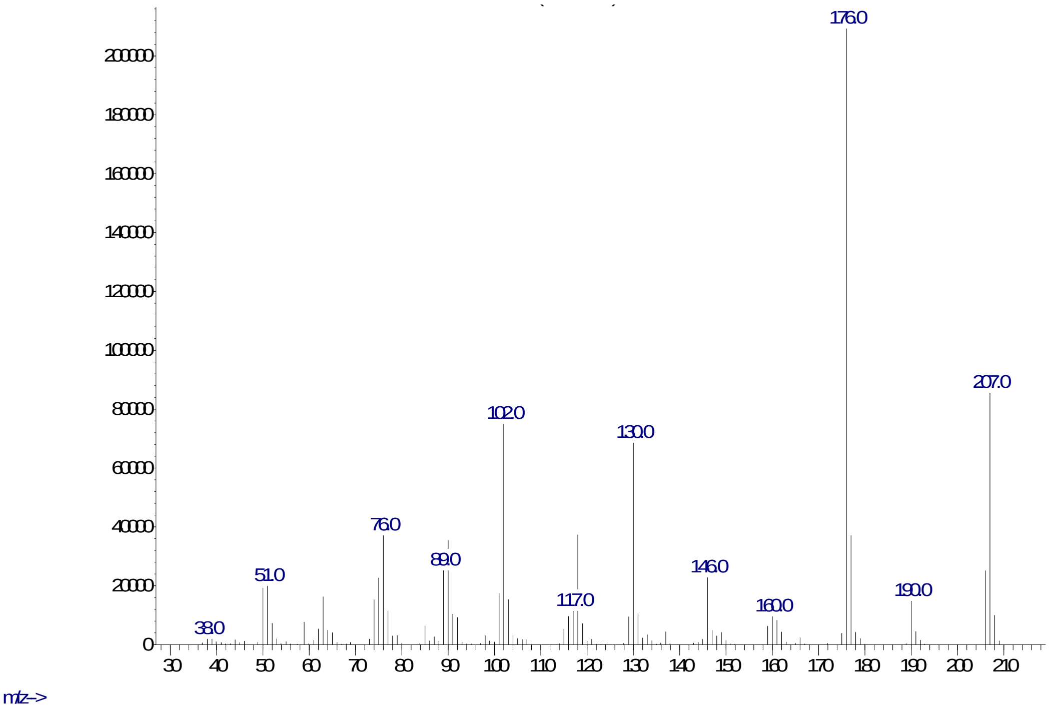
176.0
207.0
102.0
130.0
76.0
89.0
51.0
146.0
190.0
117.0
160.0
38.0
m/z-->

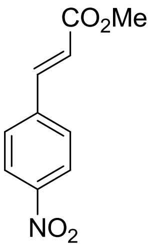
CO2Me
NO2

Methyl (*E*)-3-(3-nitrophenyl)acrylate (**3ab**):

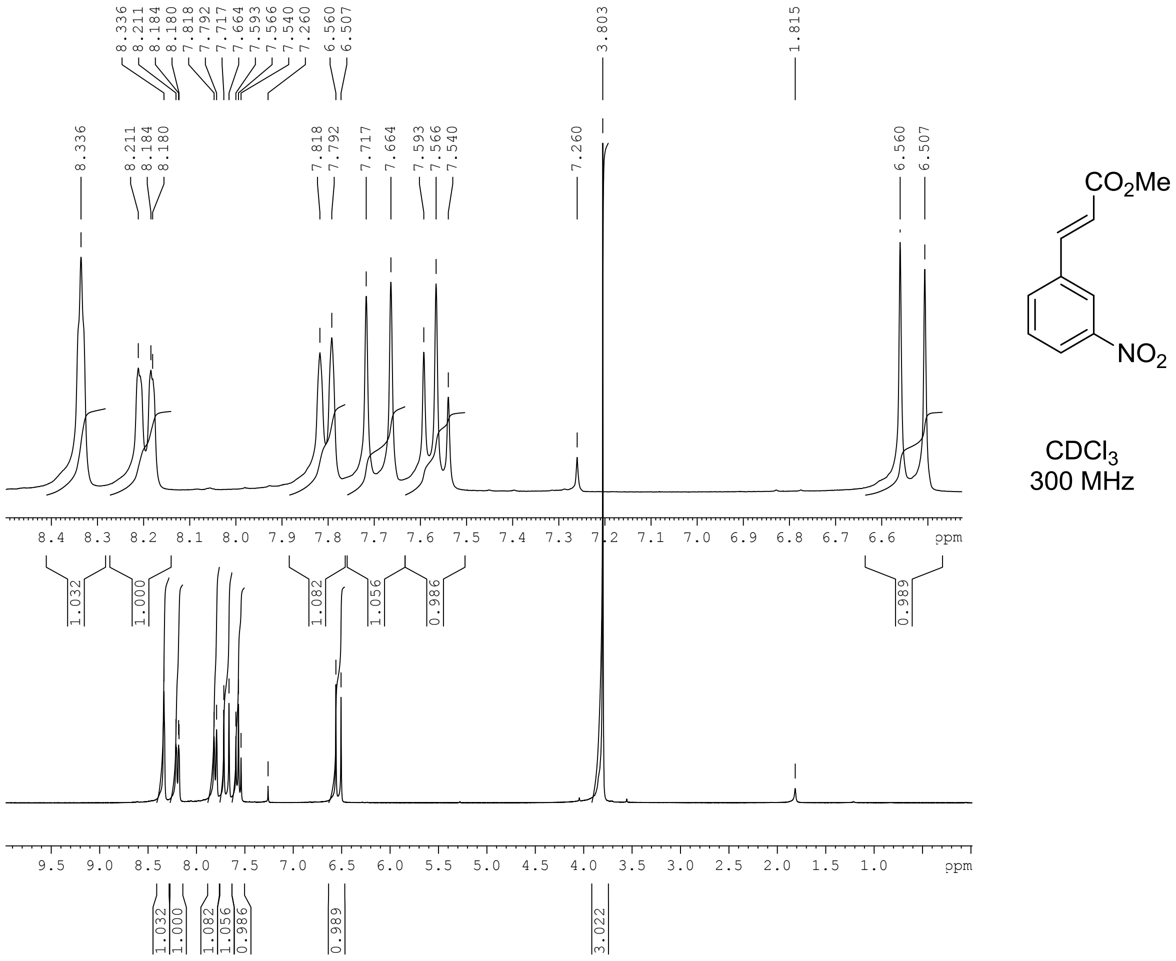

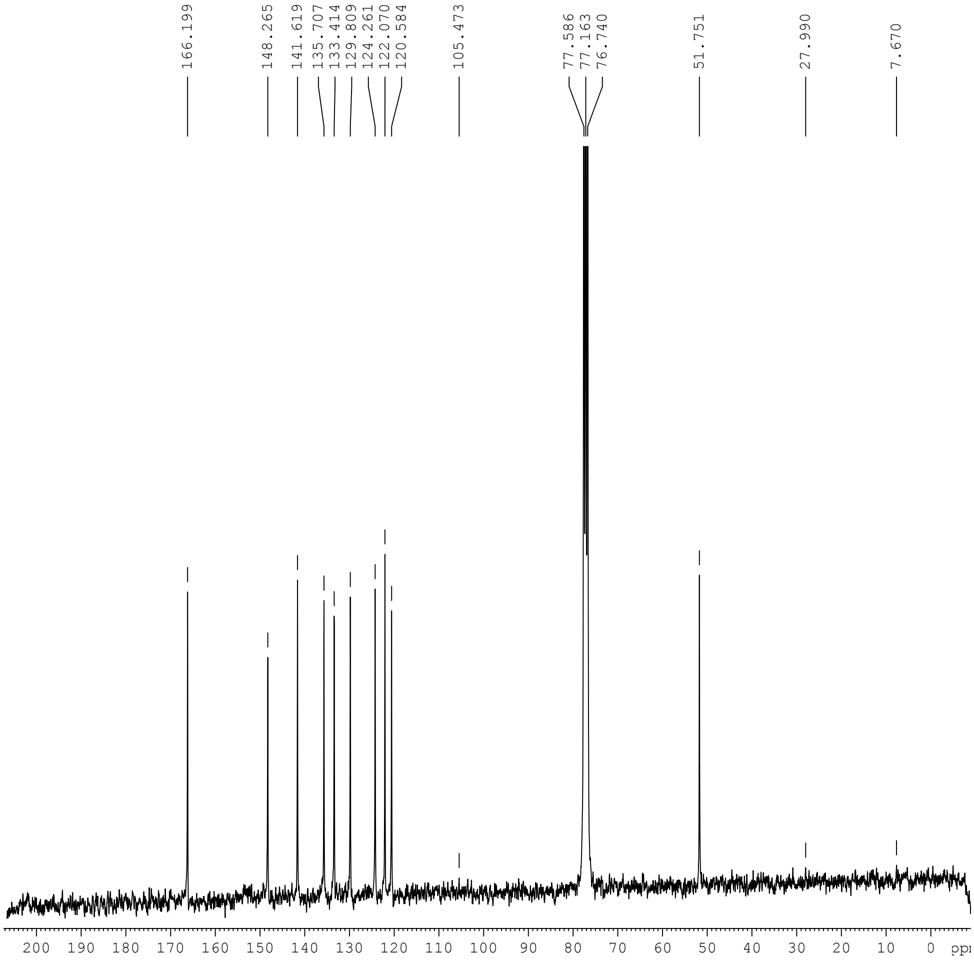
166.199
148.265
141.619
135.707
133.414
129.809
124.261
122.070
120.584
105.473
77.586
77.163
76.740
51.751
27.990
7.670
200 190 180 170 160 150 140 130 120 110 100 90 80 70 60 50 40 30 20 10 0 ppm

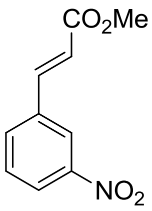
CO2Me
NO2

CDCl$_3$
75 MHz

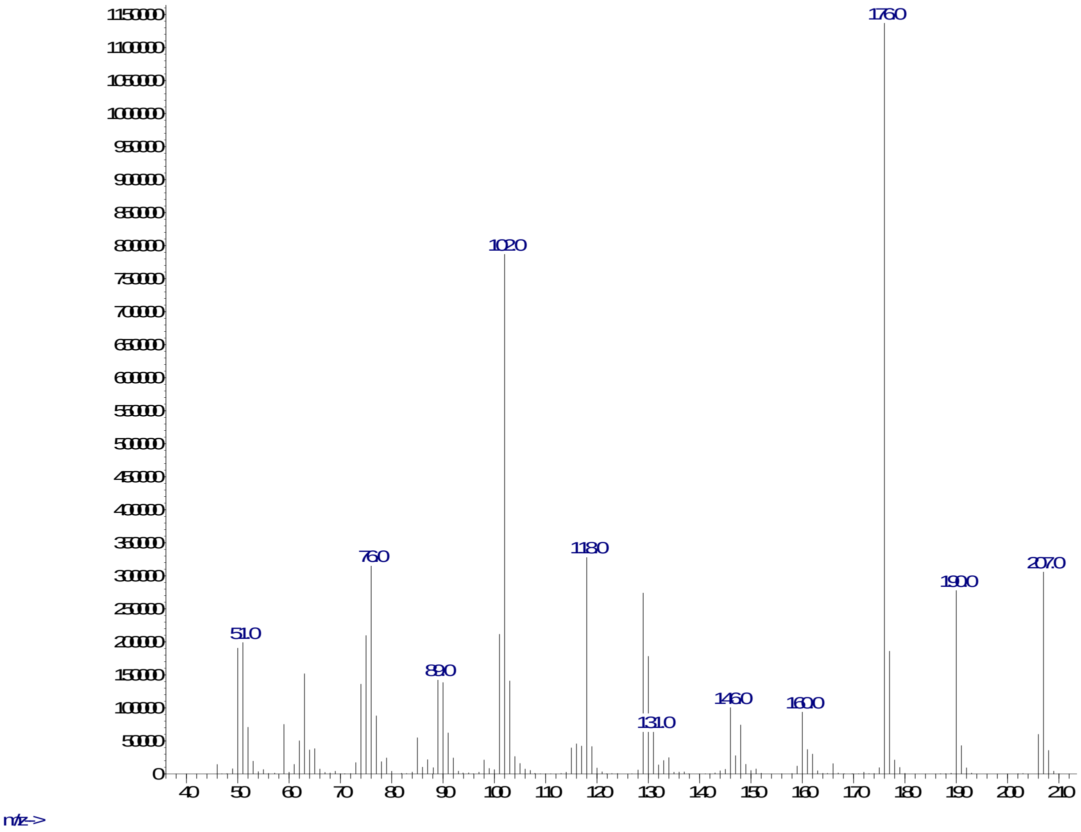
176.0
102.0
118.0
76.0
207.0
190.0
51.0
89.0
146.0
160.0
131.0
m/z-->

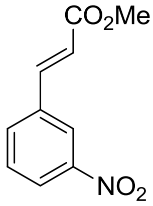
CO2Me
NO2

Methyl (*E*)-3-(2-nitrophenyl)acrylate (**3ac**):

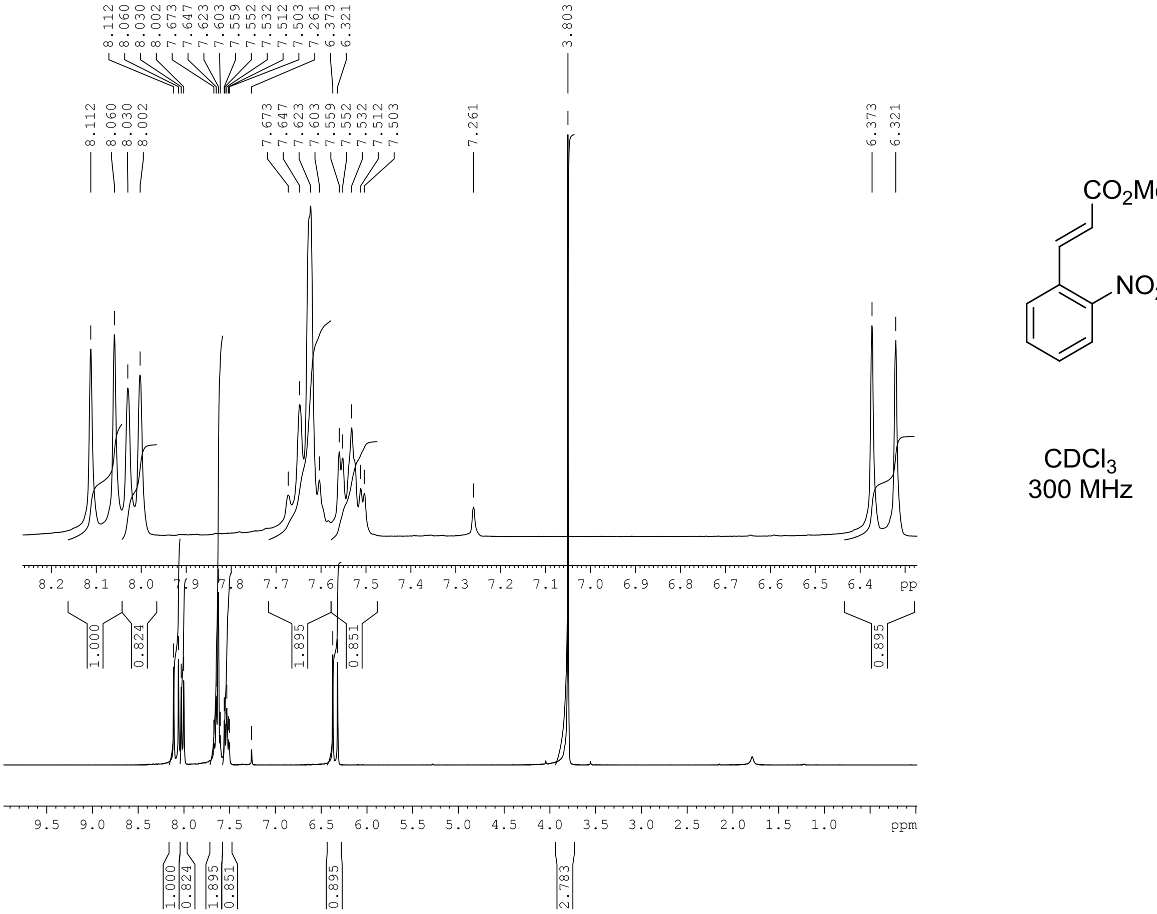

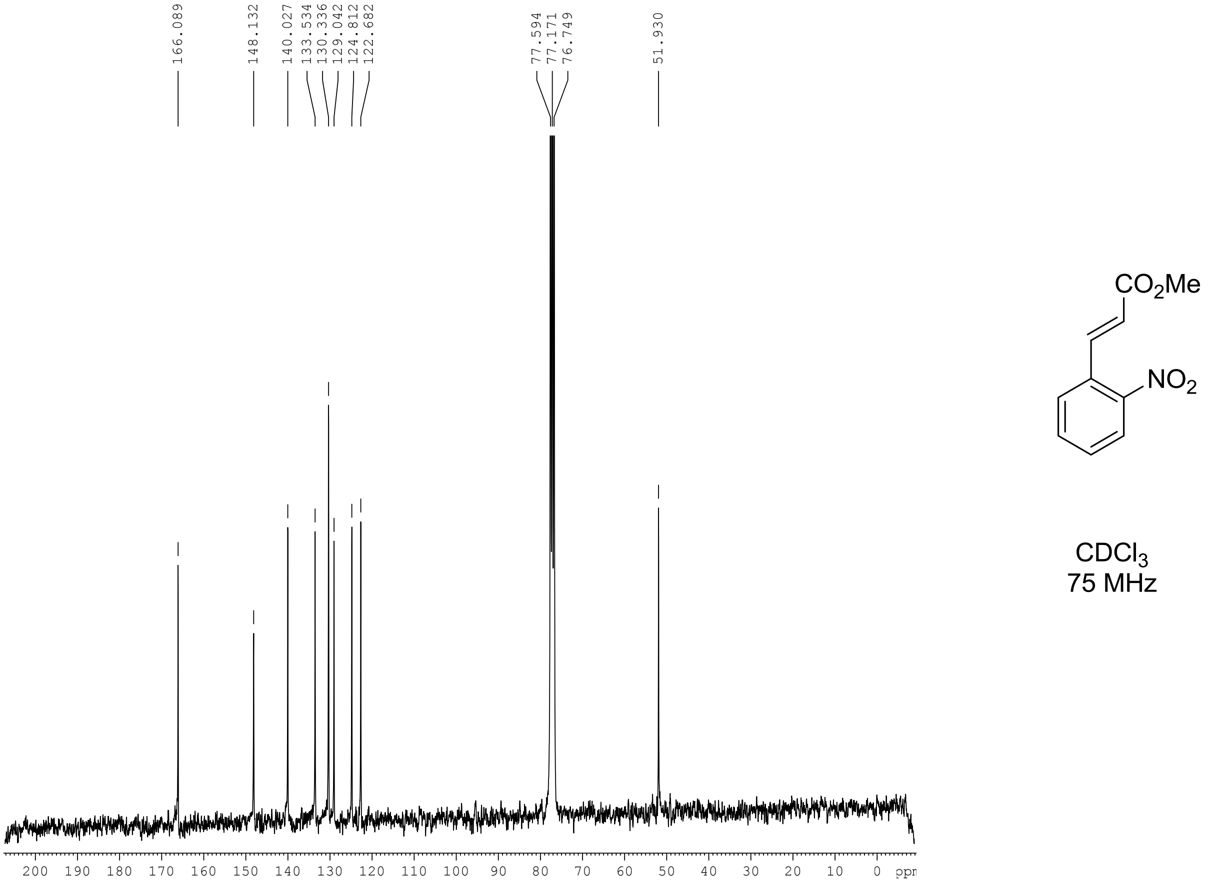

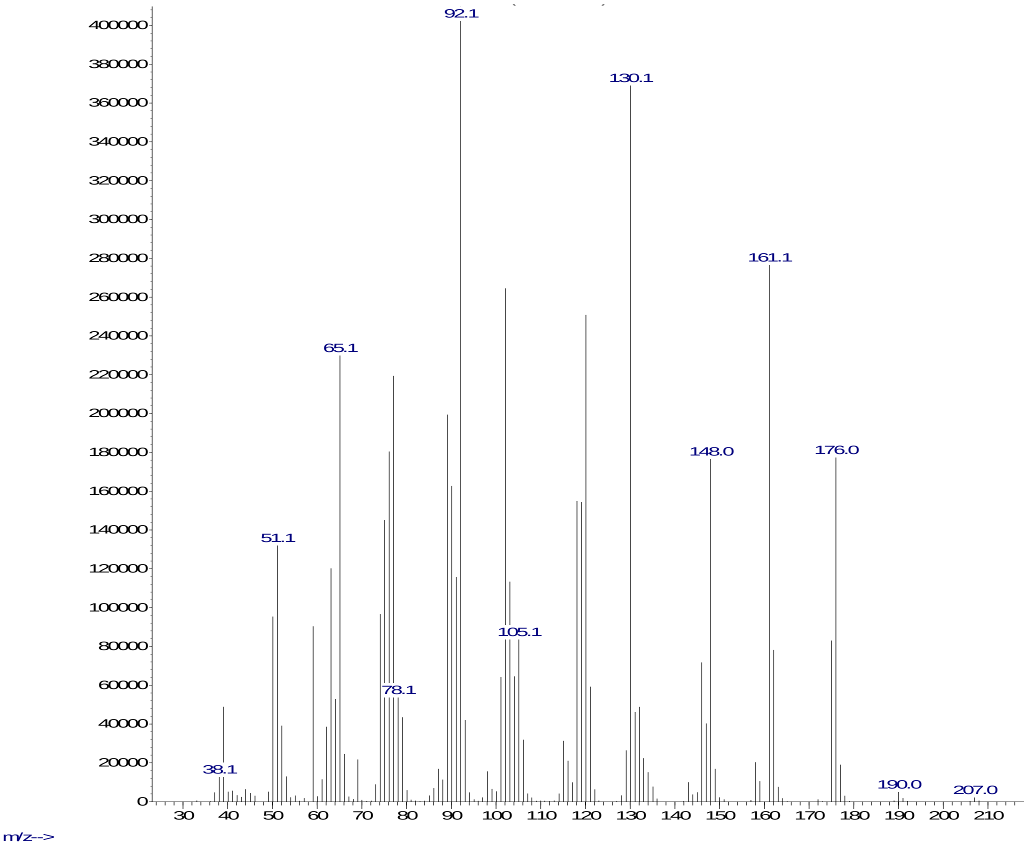
92.1
130.1
161.1
65.1
148.0
176.0
51.1
105.1
78.1
38.1
190.0
207.0
m/z-->

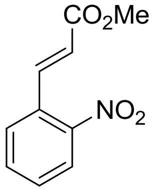
CO2Me
NO2

Methyl (*E*)-cinnamate (**3ad**):

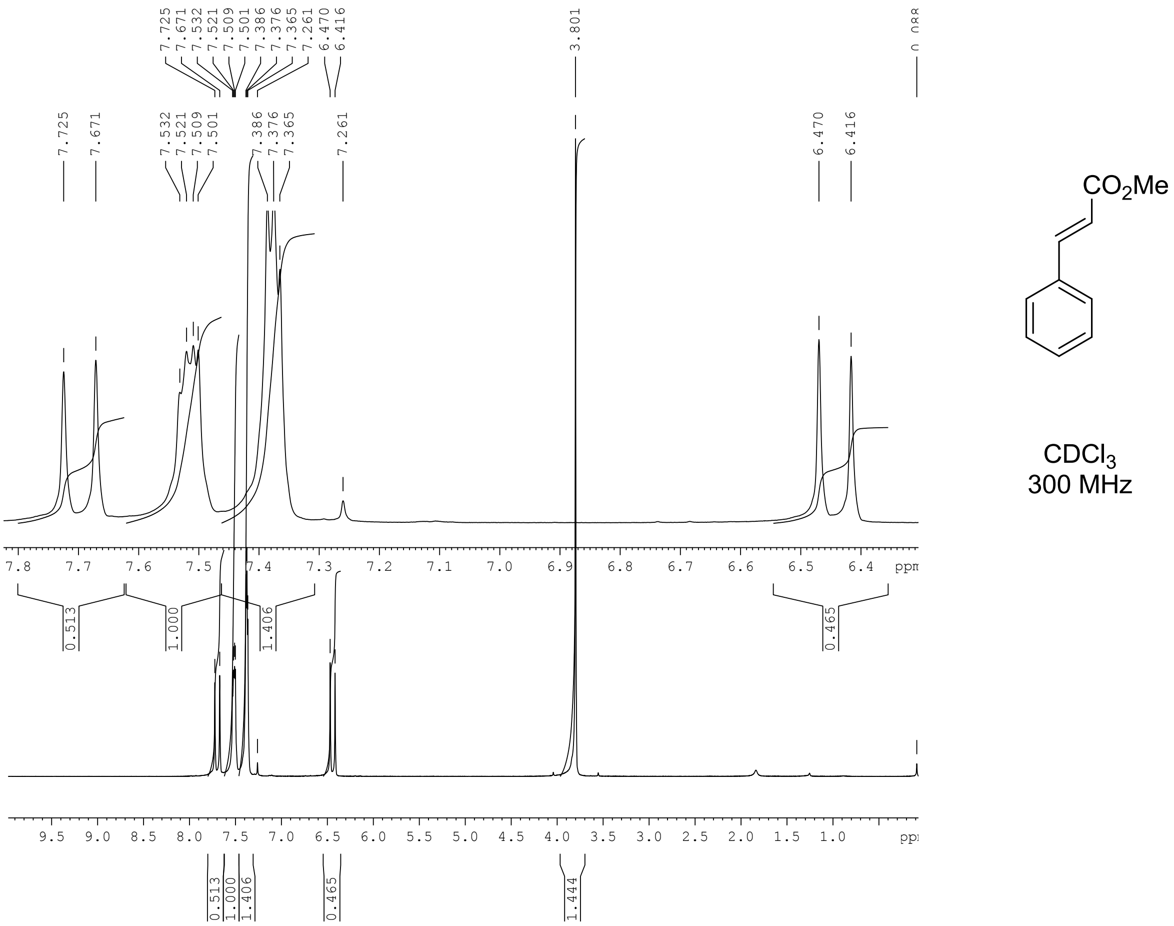

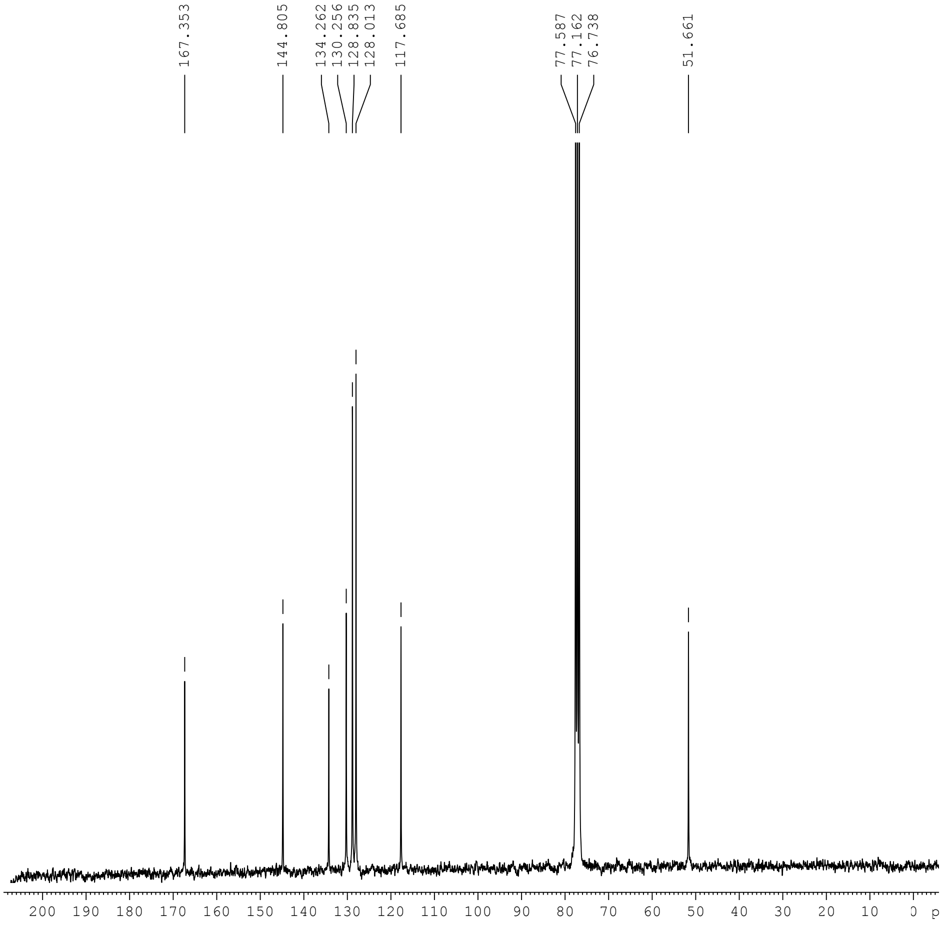
167.353
144.805
134.262
130.256
128.835
128.013
117.685
77.587
77.162
76.738
51.661
200 190 180 170 160 150 140 130 120 110 100 90 80 70 60 50 40 30 20 10 0 p

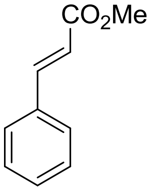
CO_2Me

$CDCl_3$
75 MHz

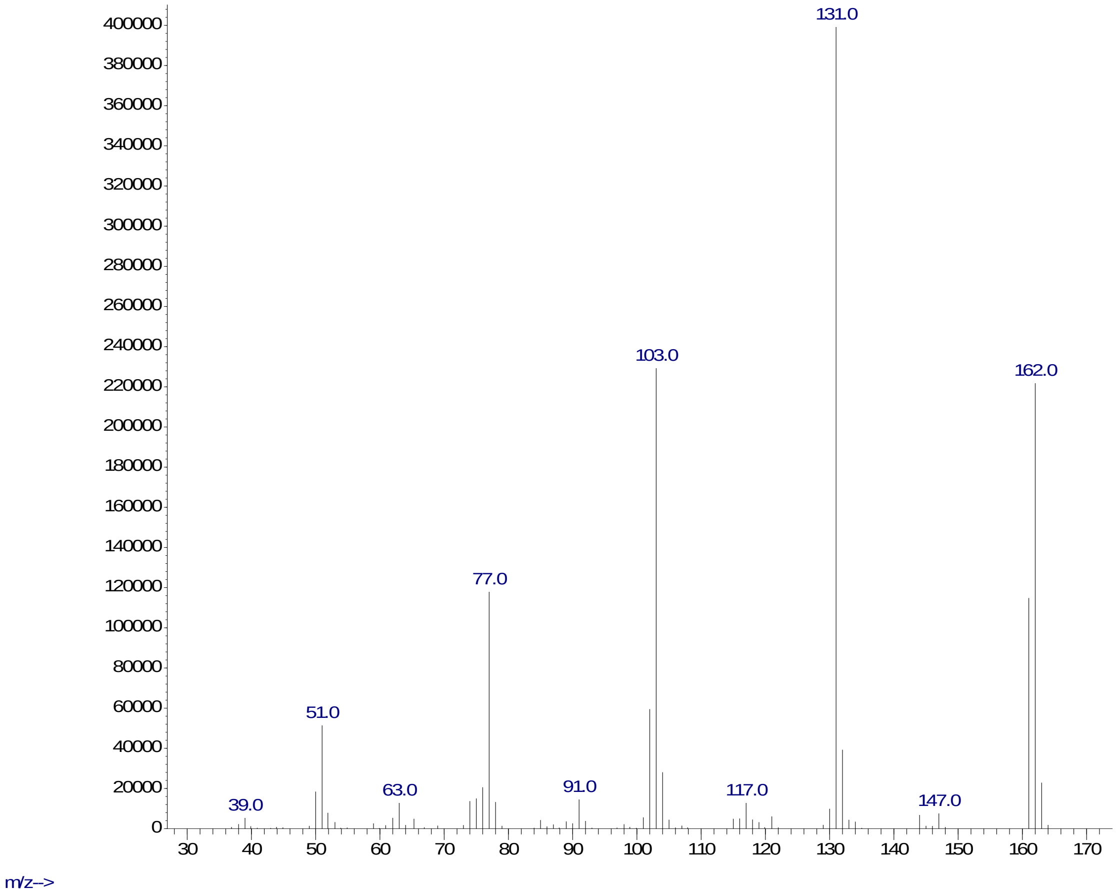
131.0
103.0
162.0
77.0
51.0
39.0
63.0
91.0
117.0
147.0
m/z-->
400000
380000
360000
340000
320000
300000
280000
260000
240000
220000
200000
180000
160000
140000
120000
100000
80000
60000
40000
20000
0
30 40 50 60 70 80 90 100 110 120 130 140 150 160 170

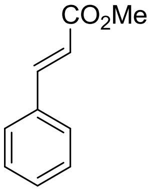
CO_2Me

Methyl (*E*)-3-(4-methoxyphenyl)acrylate (**3ae**):

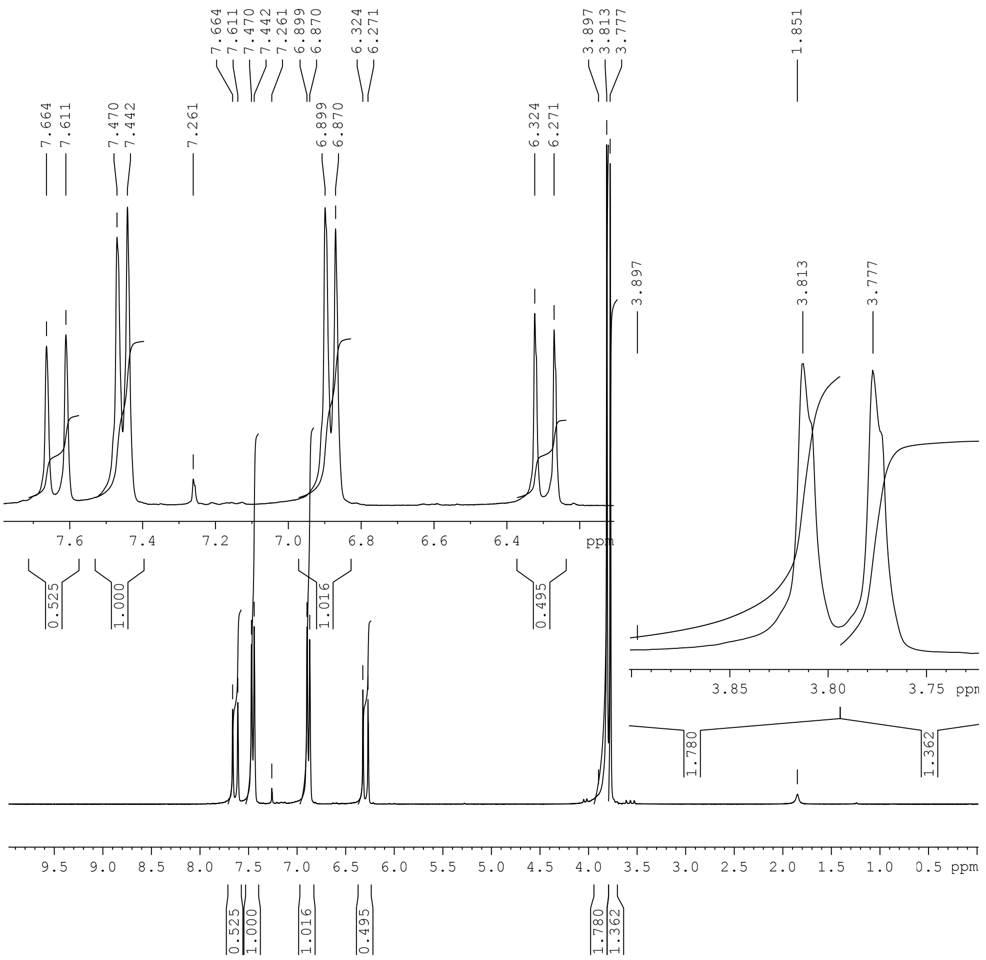

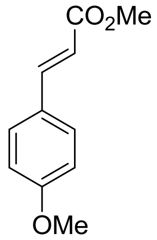

$CDCl_3$
300 MHz

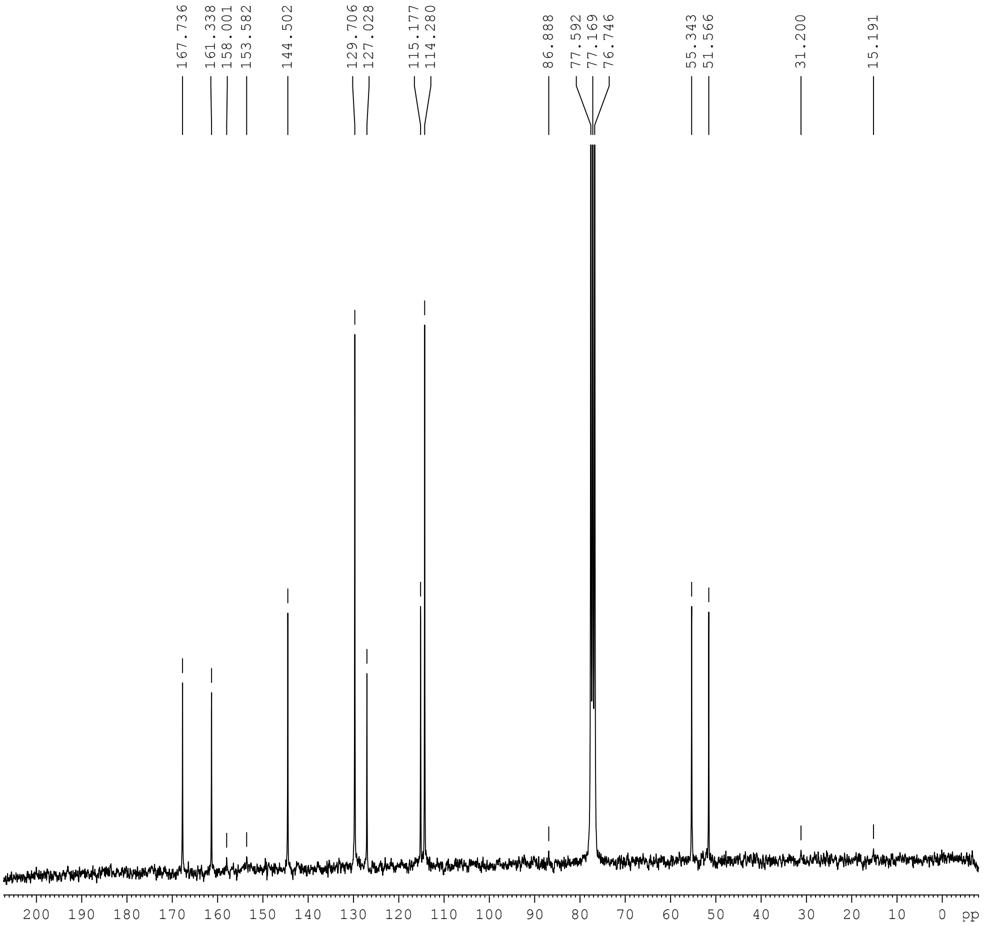

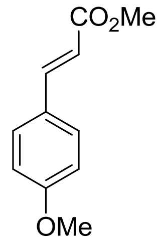

$CDCl_3$
75 MHz

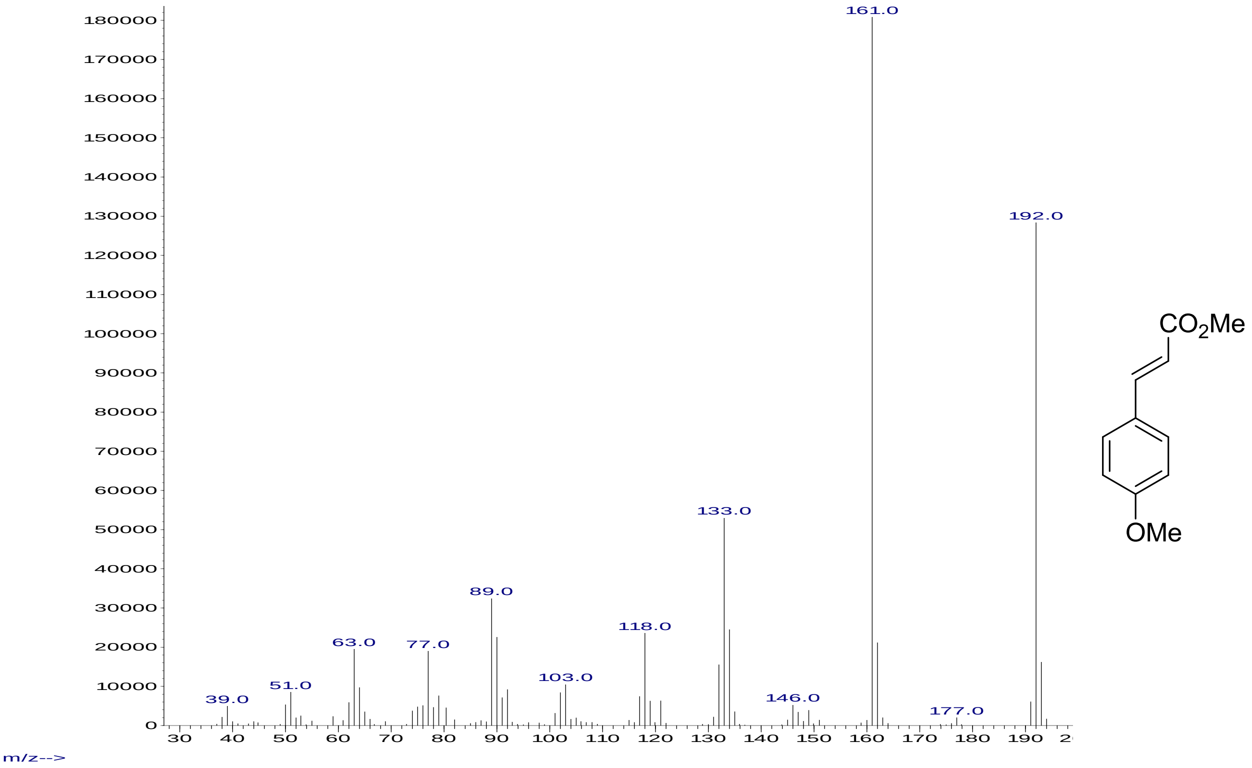

Methyl (*E*)-4-(3-methoxy-3-oxoprop-1-en-1-yl)benzoate (**3af**):

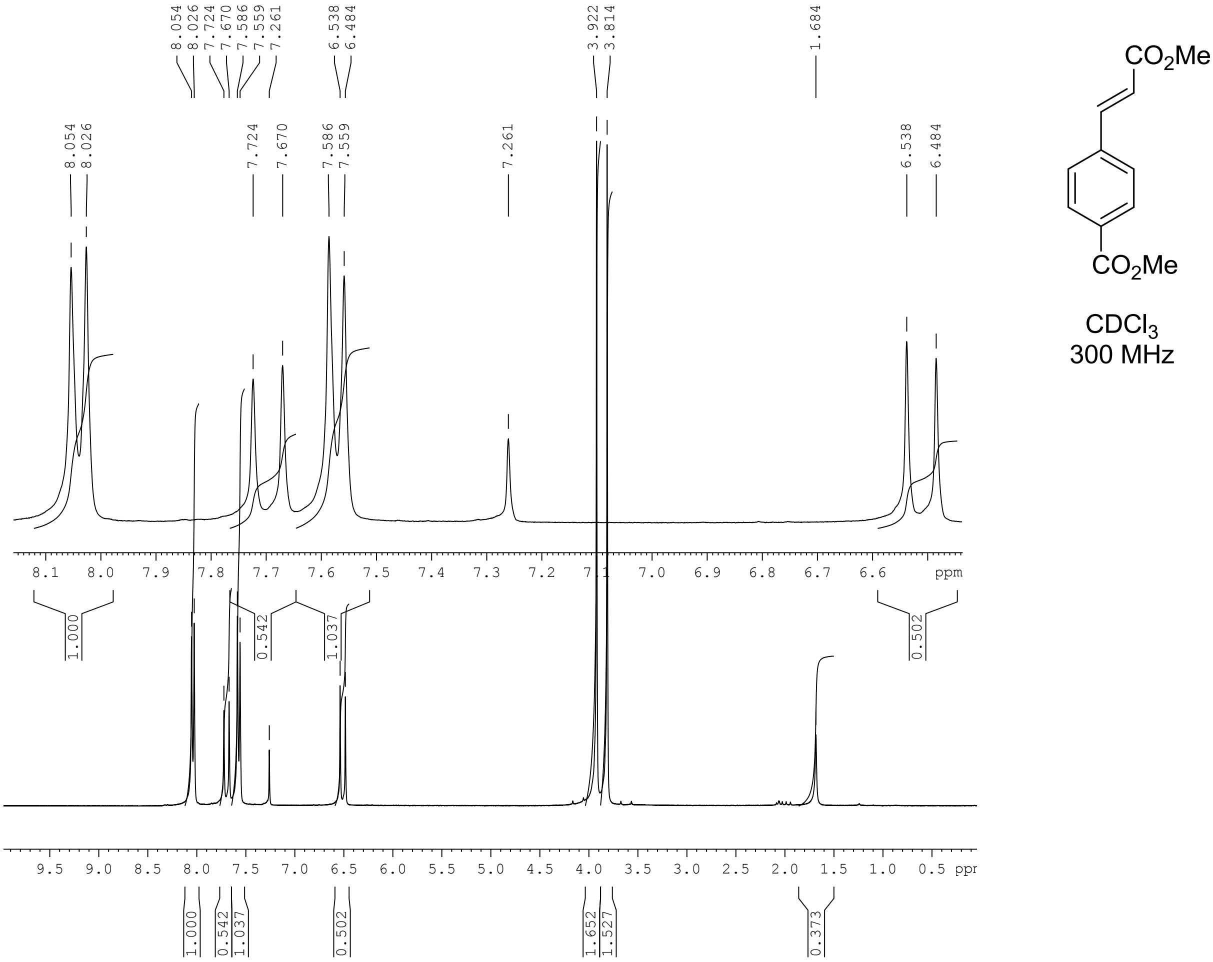

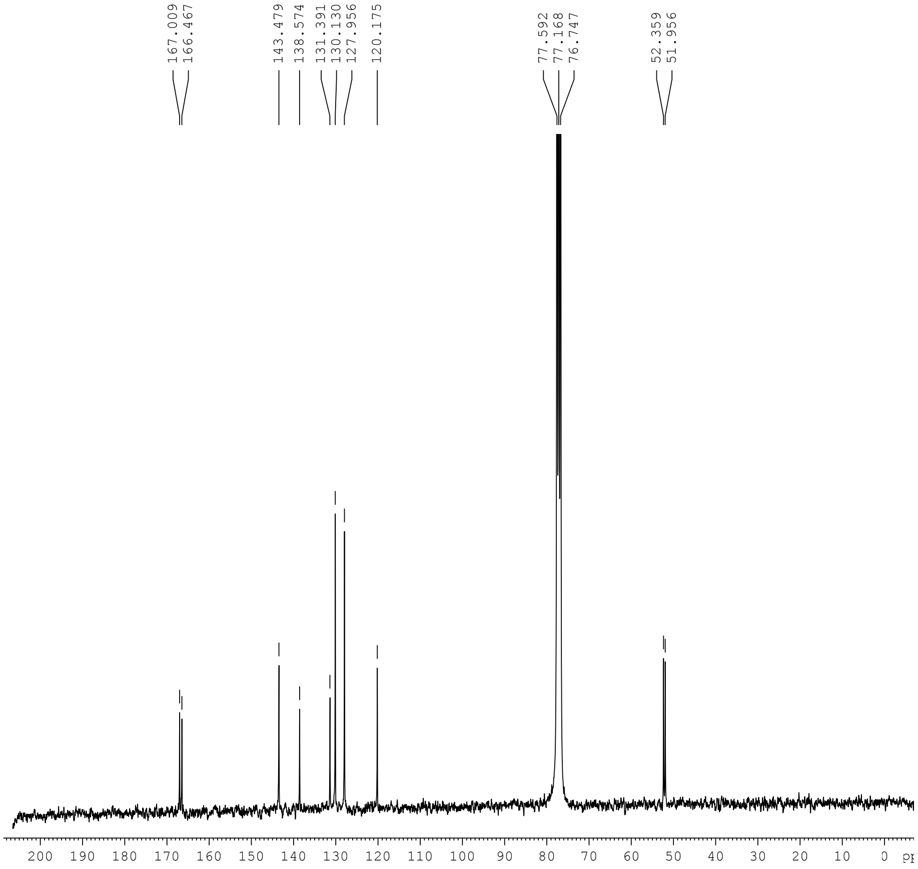
167.009
166.467
143.479
138.574
131.391
130.130
127.956
120.175
77.592
77.168
76.747
52.359
51.956
200 190 180 170 160 150 140 130 120 110 100 90 80 70 60 50 40 30 20 10 0 ppm

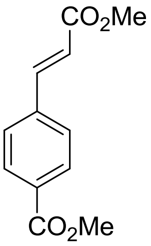
CO_2Me
CO_2Me

$CDCl_3$
75 MHz

Methyl (*E*)-3-(4-cyanophenyl)acrylate (**3ag**):

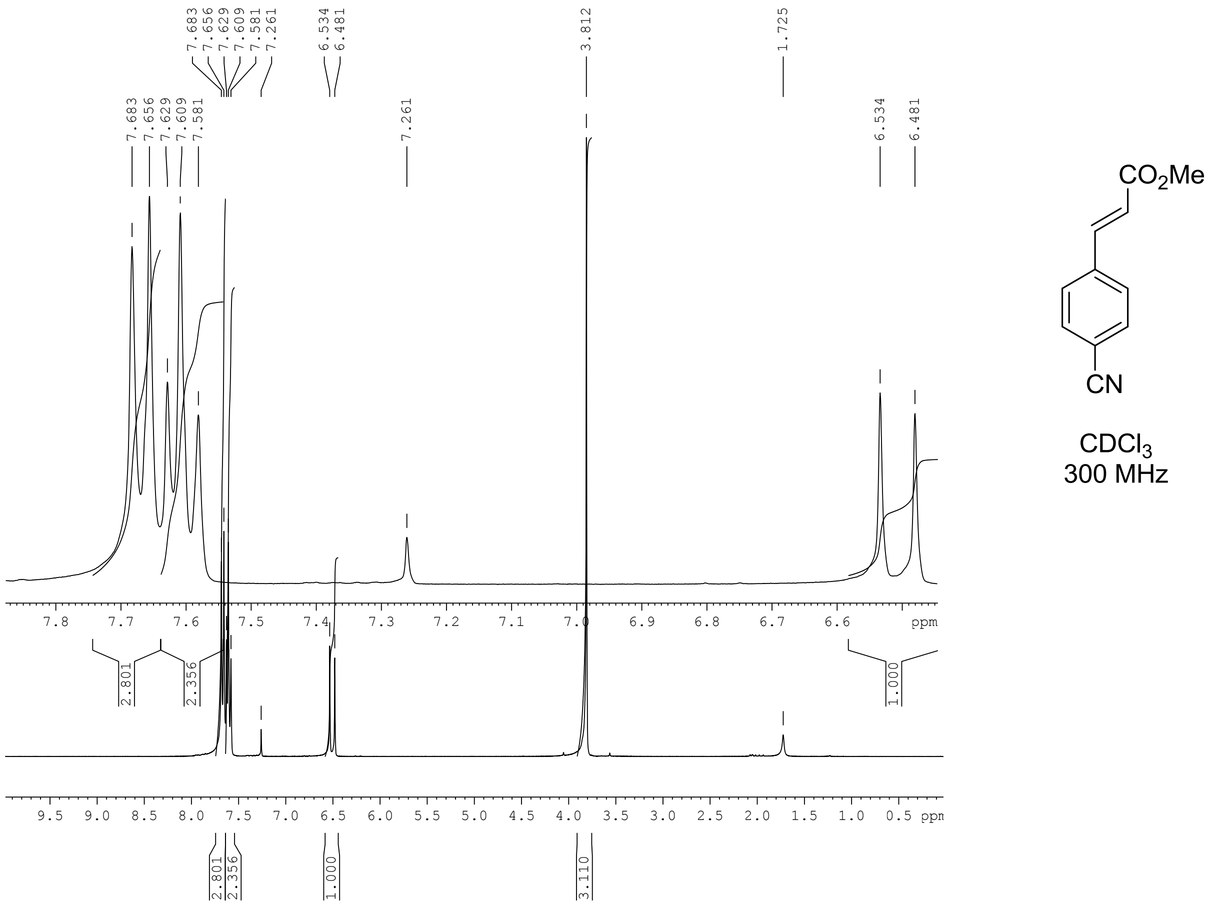

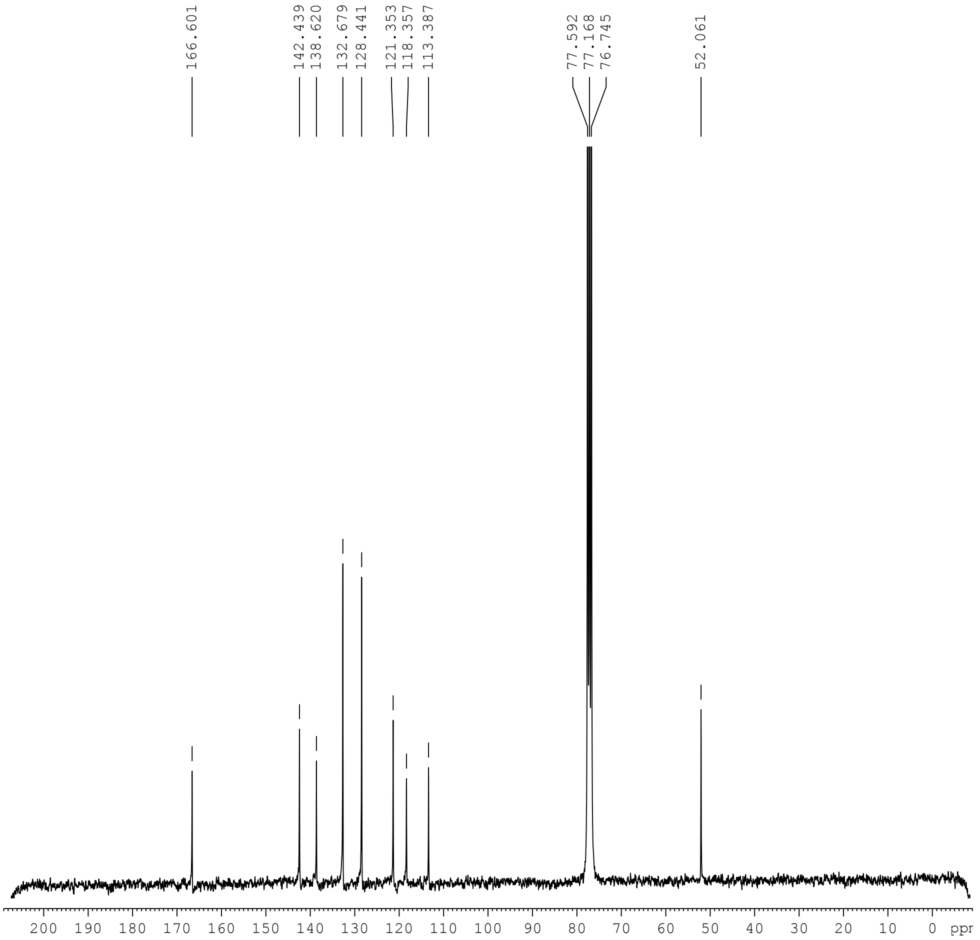
166.601
142.439
138.620
132.679
128.441
121.353
118.357
113.387
77.592
77.168
76.745
52.061
200 190 180 170 160 150 140 130 120 110 100 90 80 70 60 50 40 30 20 10 0 ppm

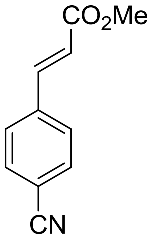
CO_2Me
CN

$CDCl_3$
75 MHz

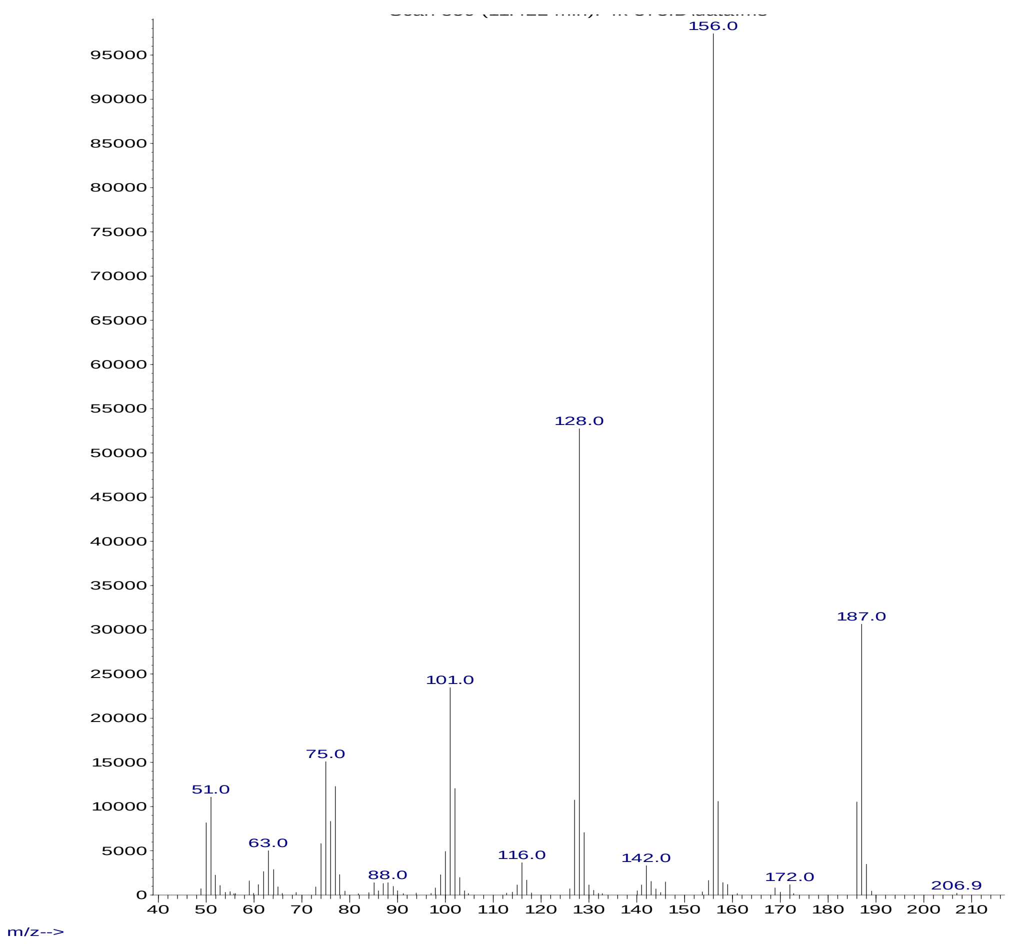
156.0
128.0
187.0
101.0
75.0
51.0
63.0
88.0
116.0
142.0
172.0
206.9
m/z-->

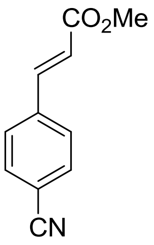
CO2Me
CN

Methyl (*E*)-3-(4-bromophenyl)acrylate (**3ah**):

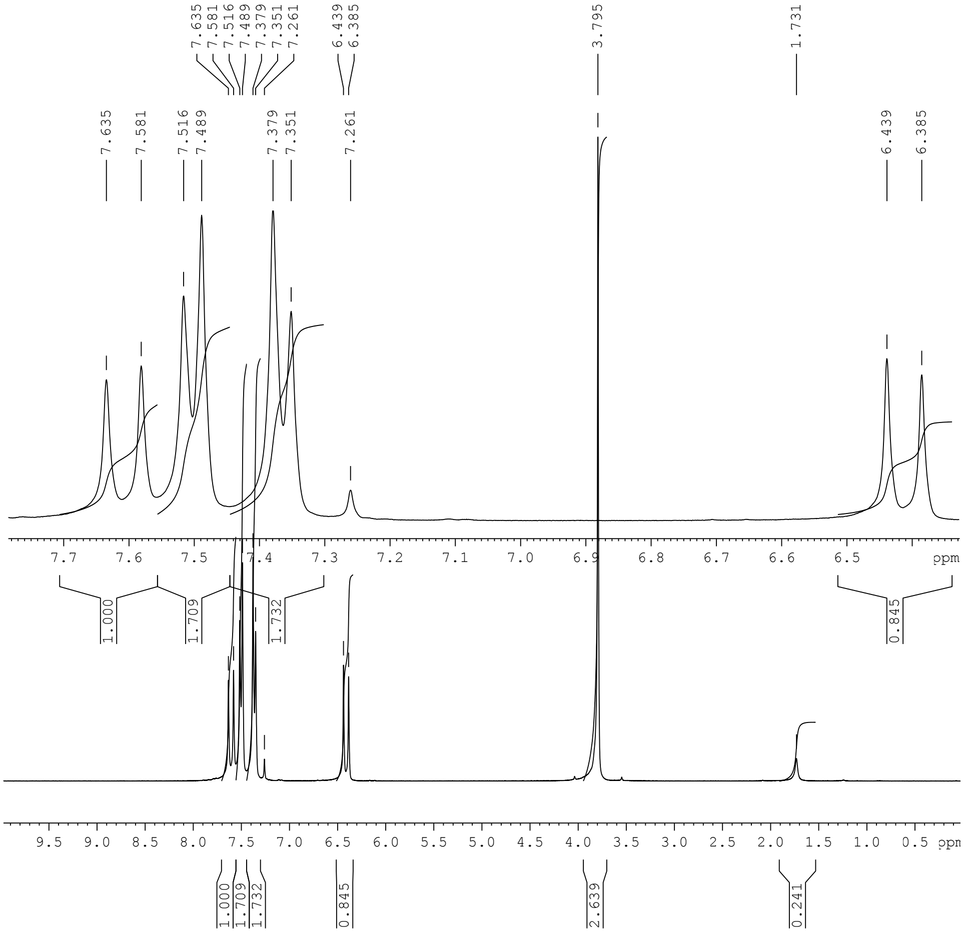

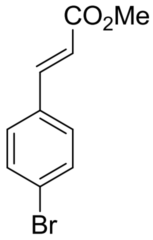

$CDCl_3$
300 MHz

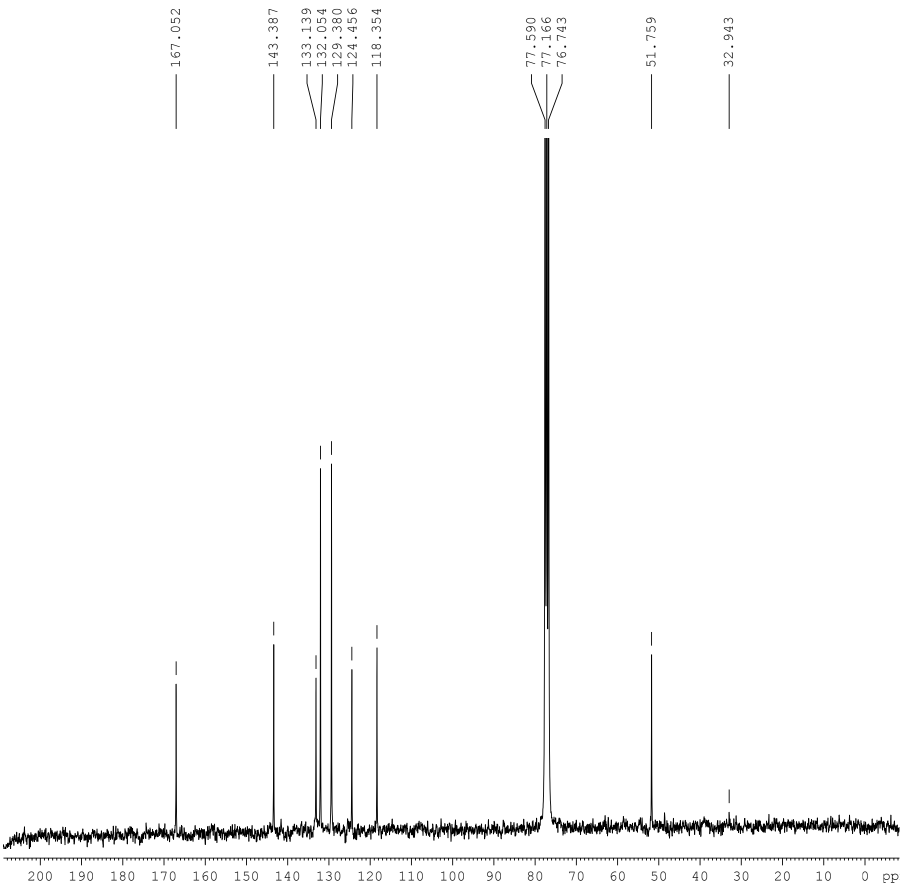
167.052
143.387
133.139
132.054
129.380
124.456
118.354
77.590
77.166
76.743
51.759
32.943
200 190 180 170 160 150 140 130 120 110 100 90 80 70 60 50 40 30 20 10 0 pp

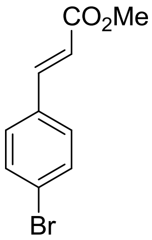
CO2Me
Br

$CDCl_3$
75 MHz

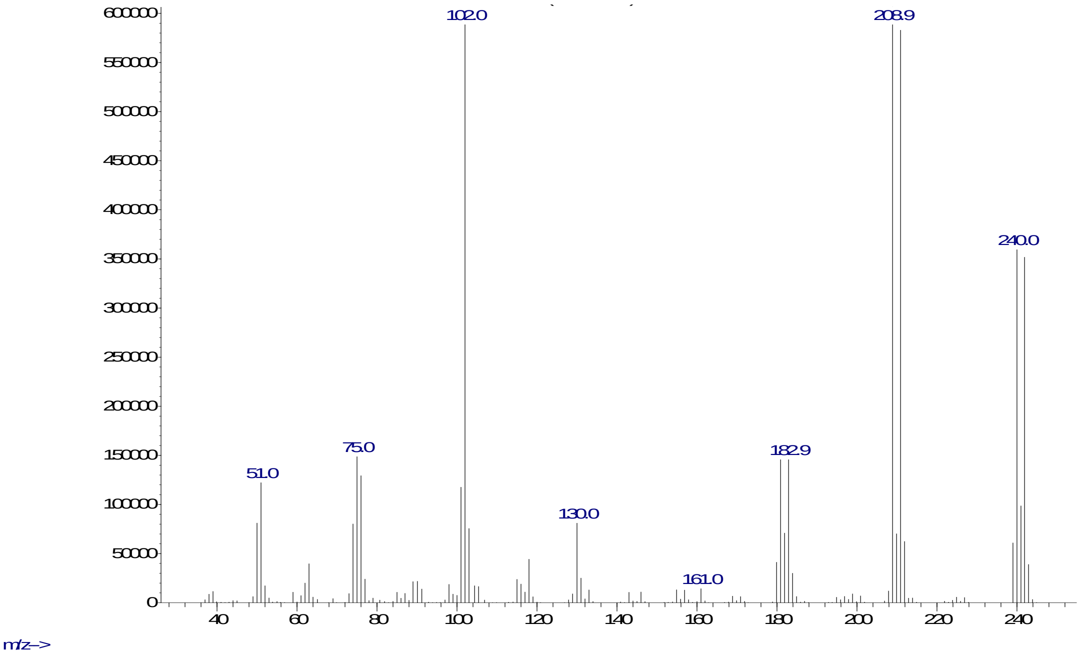
600000
550000
500000
450000
400000
350000
300000
250000
200000
150000
100000
50000
0
102.0
208.9
240.0
51.0
75.0
130.0
161.0
182.9
40 60 80 100 120 140 160 180 200 220 240
m/z-->

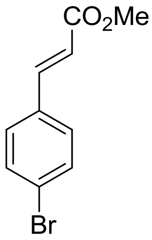
CO2Me
Br

Methyl (*E*)-3-(2-methoxyphenyl)acrylate (**3ai**):

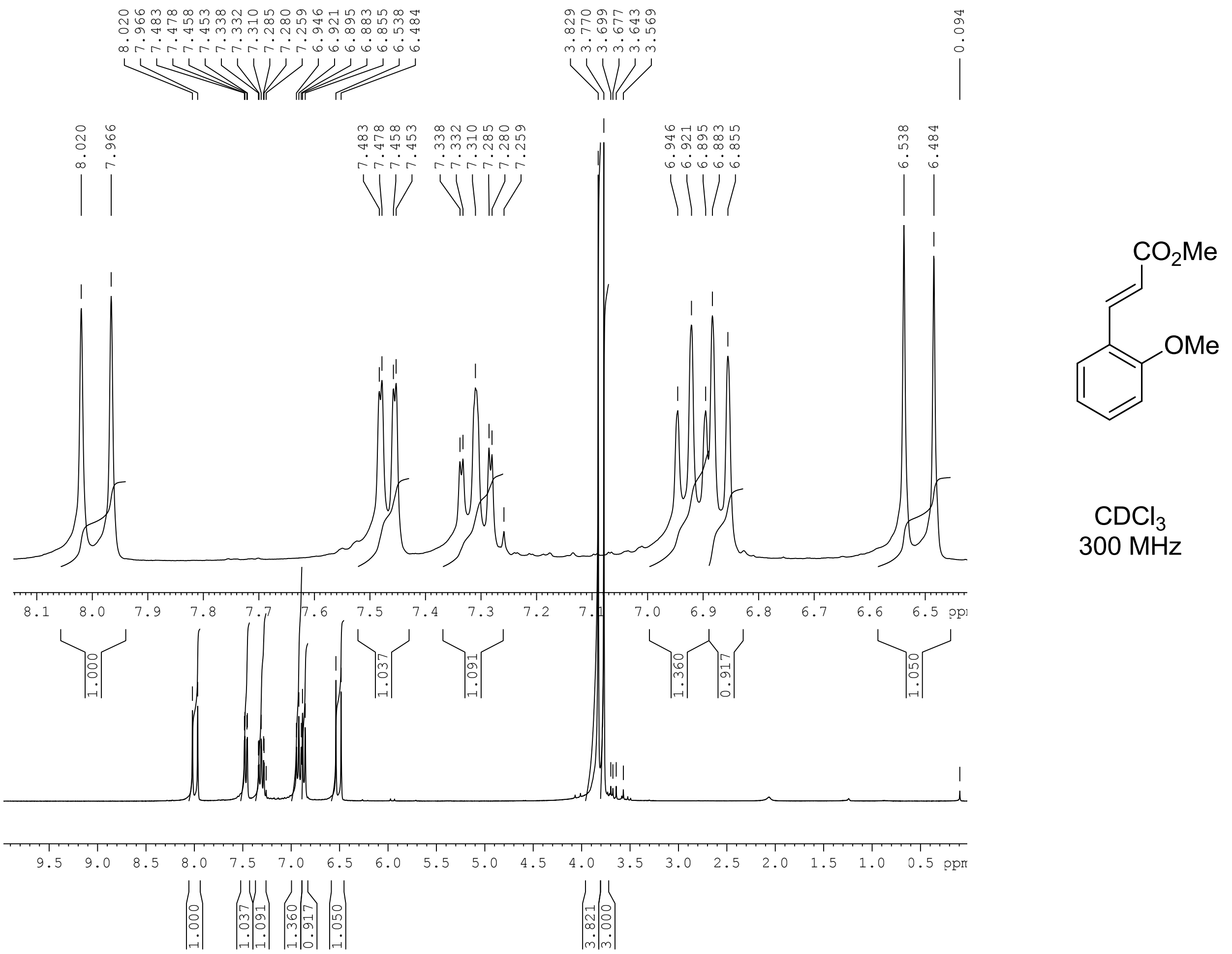

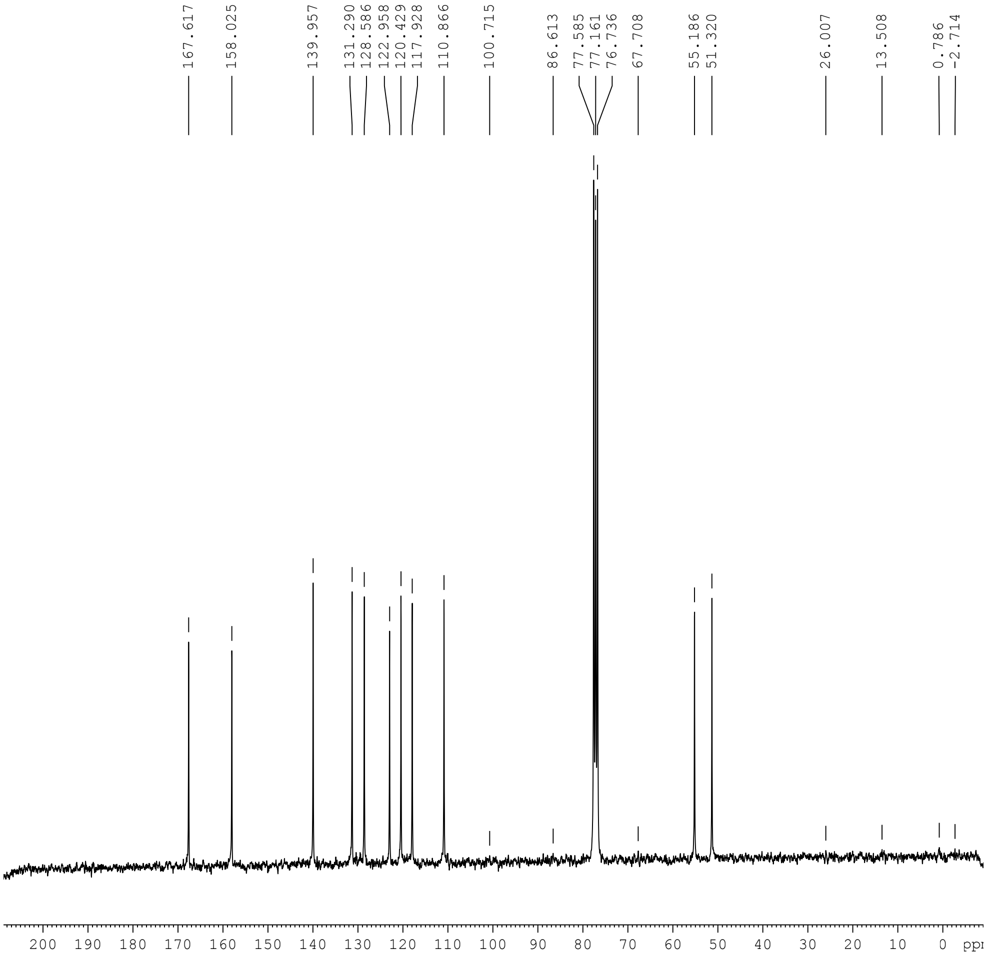
167.617
158.025
139.957
131.290
128.586
122.958
120.429
117.928
110.866
100.715
86.613
77.585
77.161
76.736
67.708
55.186
51.320
26.007
13.508
0.786
-2.714
ppm

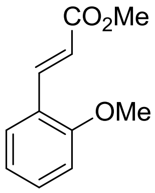
CO2Me
OMe

$CDCl_3$
75 MHz

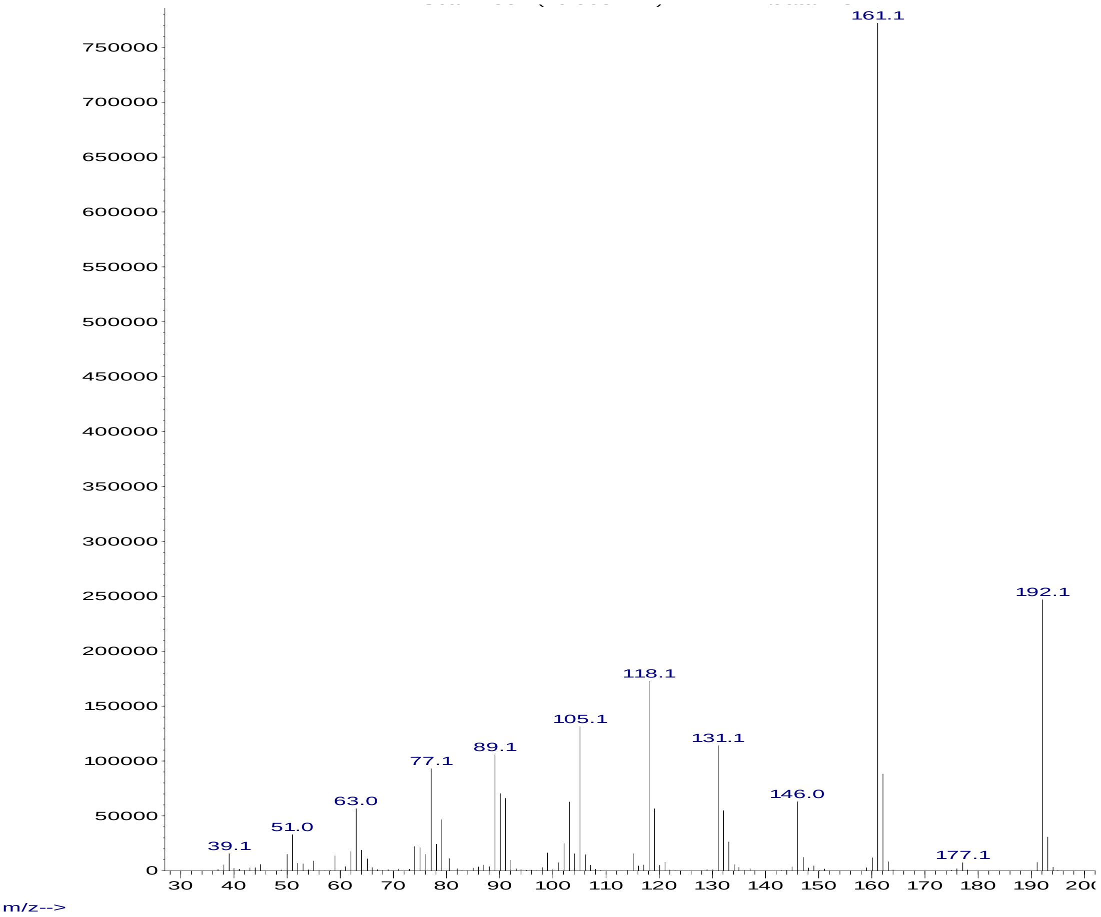
161.1
192.1
118.1
105.1
131.1
89.1
77.1
146.0
63.0
51.0
39.1
177.1
m/z-->

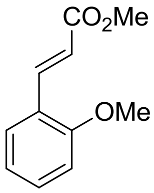
CO2Me
OMe

3-Chloropropyl (*E*)-3-(4-nitrophenyl)acrylate (**3ba**):

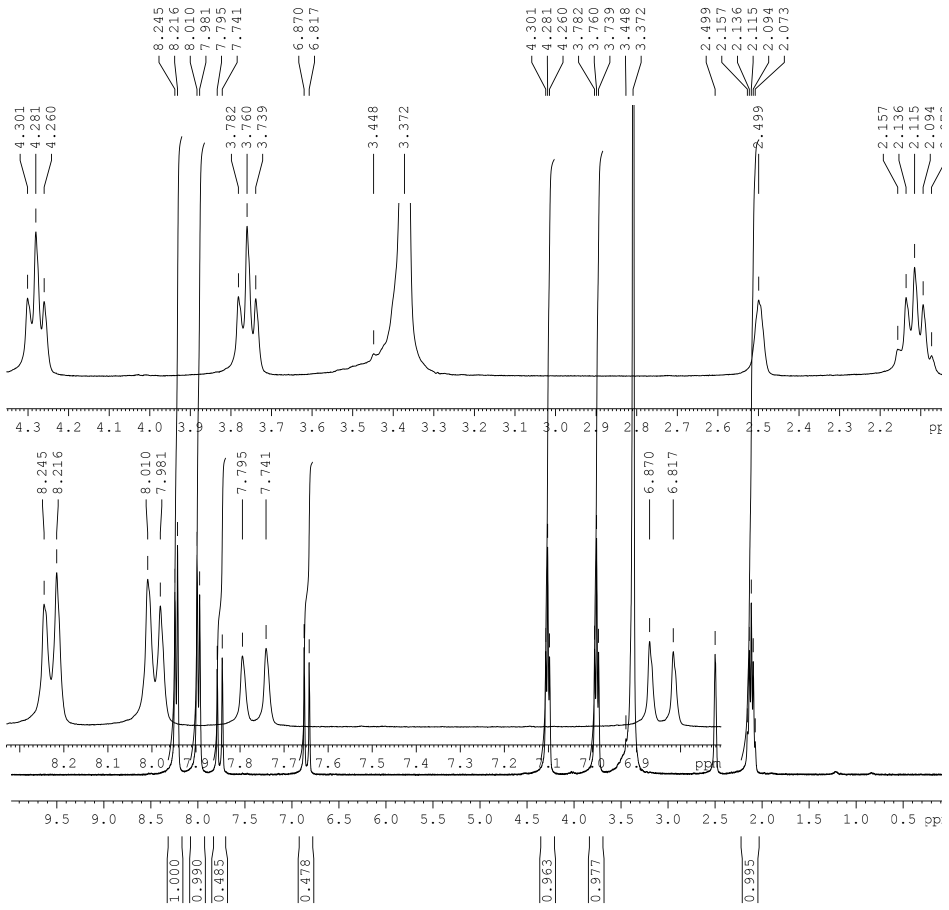

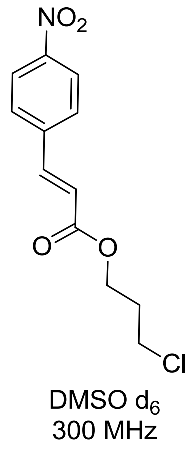

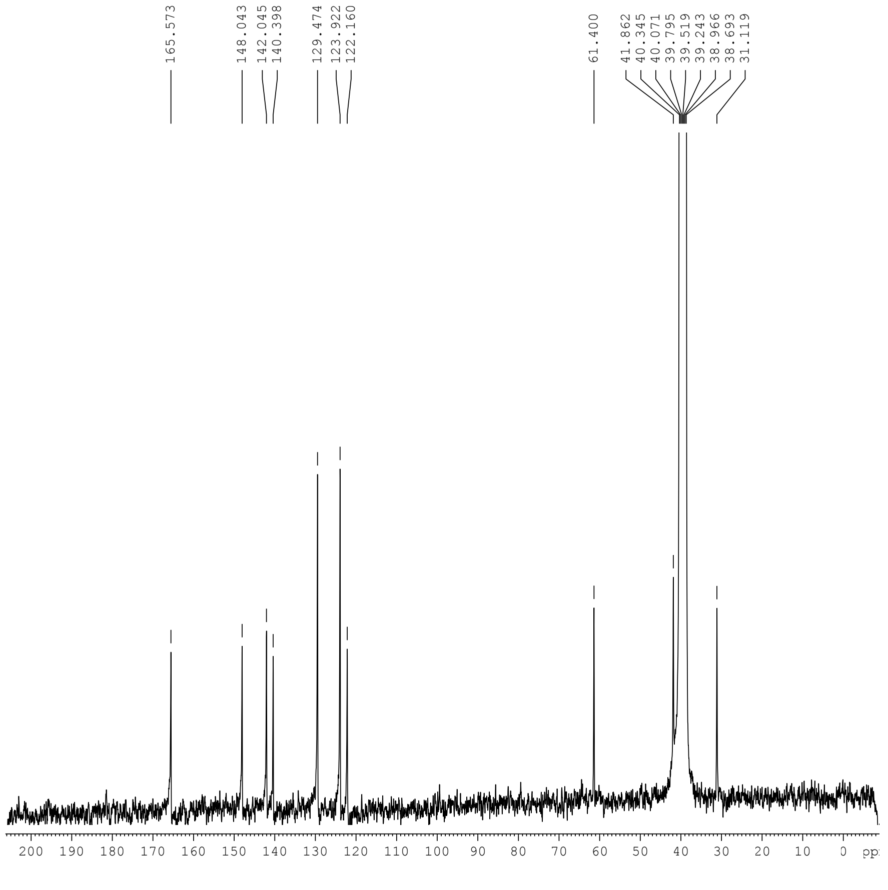

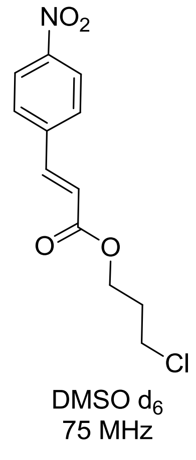

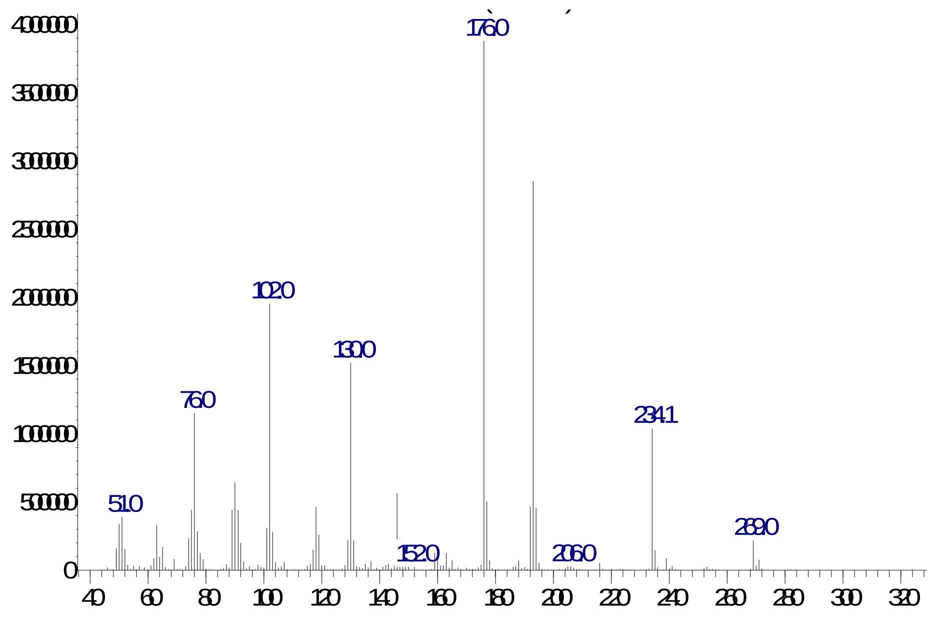

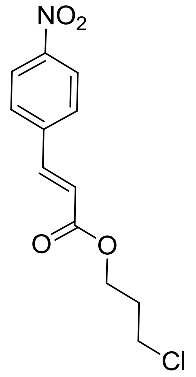

3-Chloropropyl (*E*)-3-(3-nitrophenyl)acrylate (**3bb**):

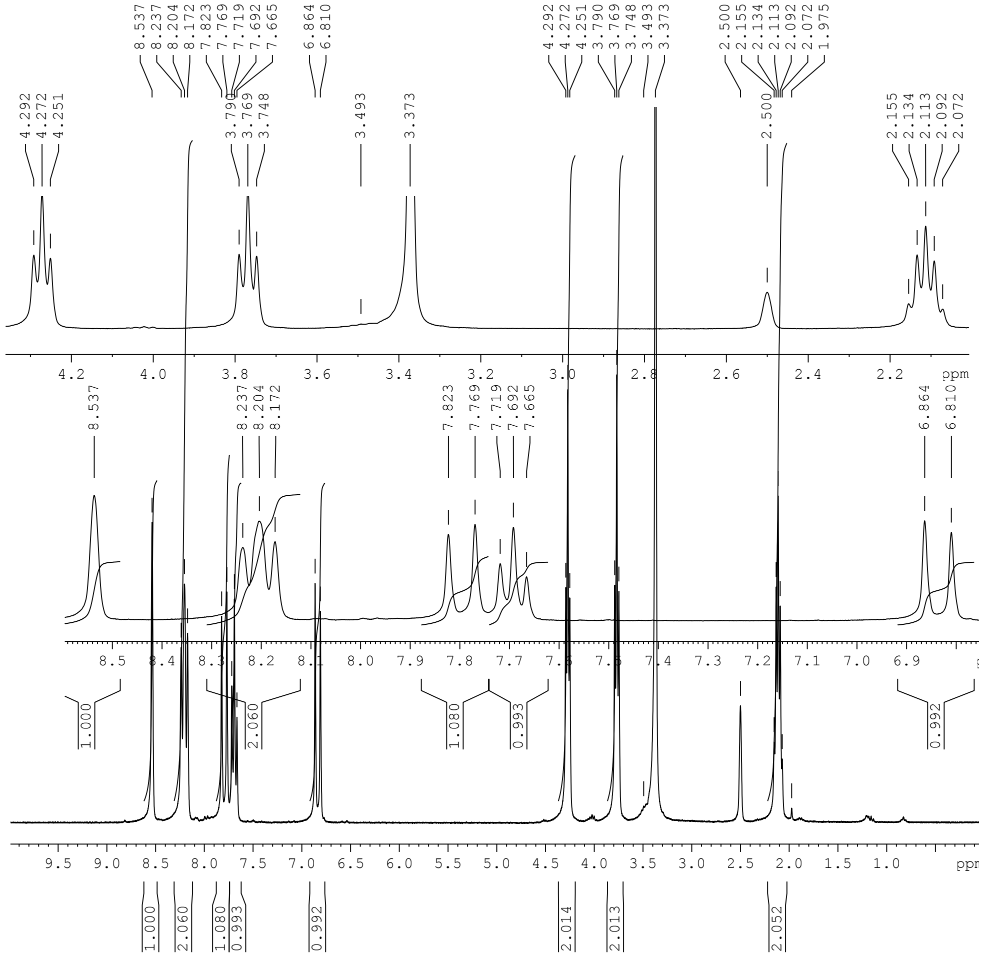

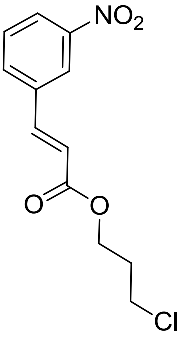

DMSO d_6
300 MHz

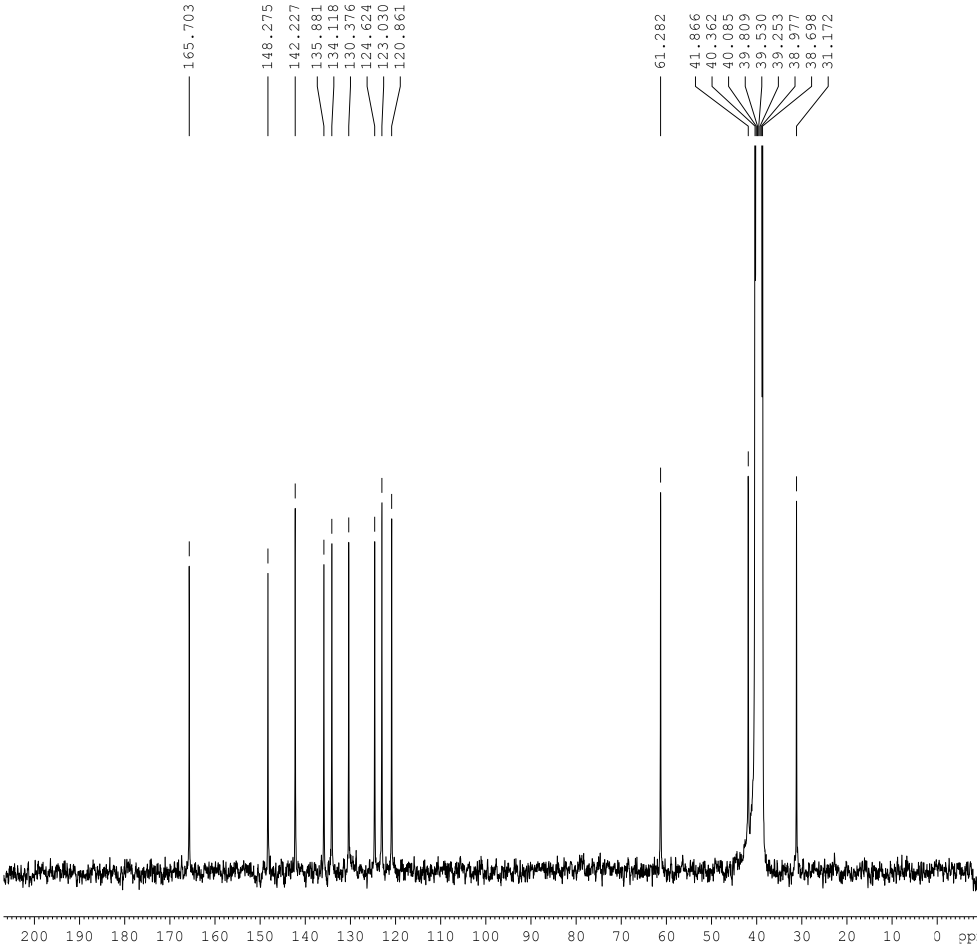

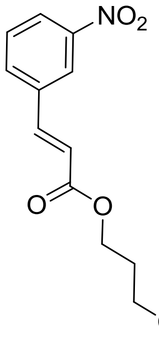

DMSO d_6
75 MHz

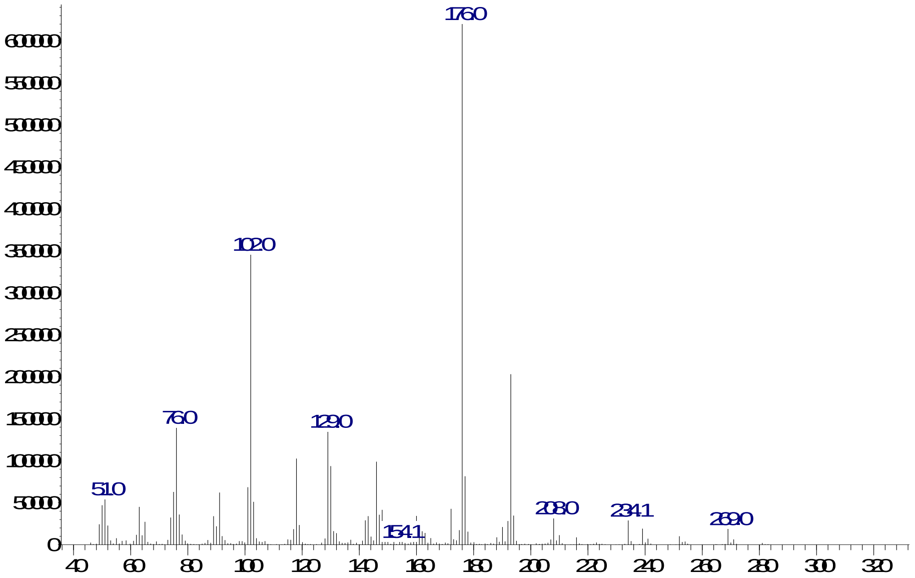

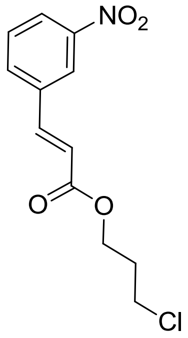

3-Chloropropyl (E)-3-(4-cyanophenyl)acrylate (**3bg**):

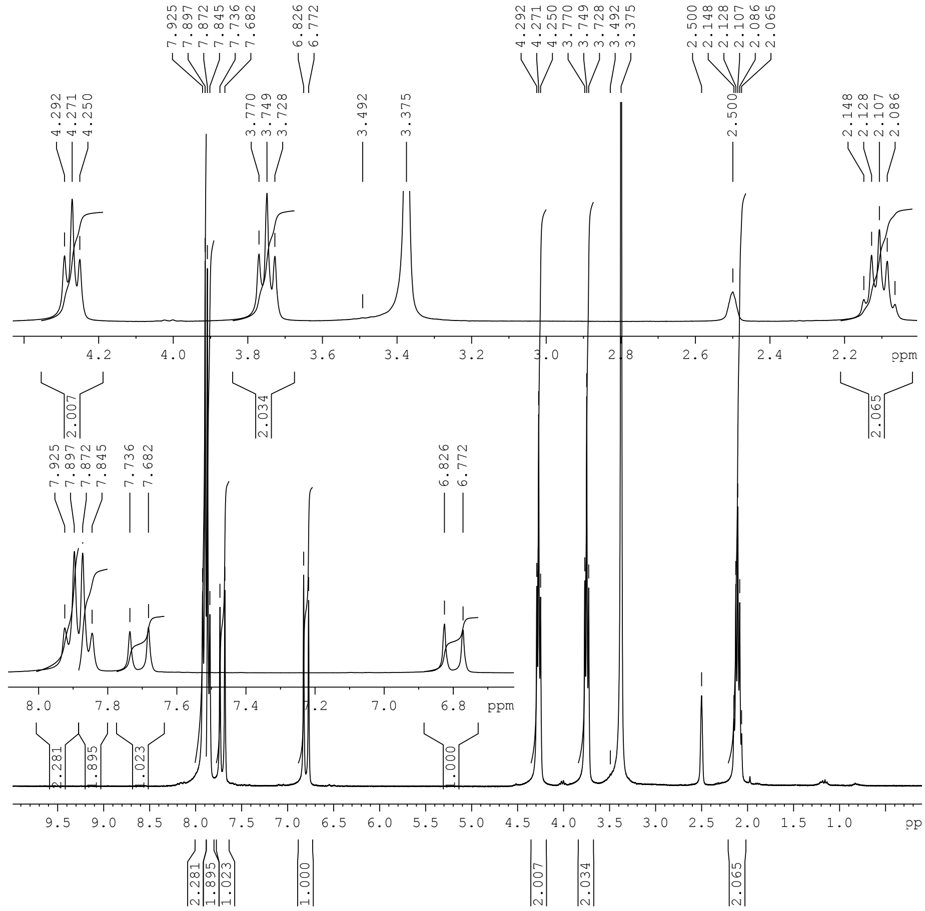

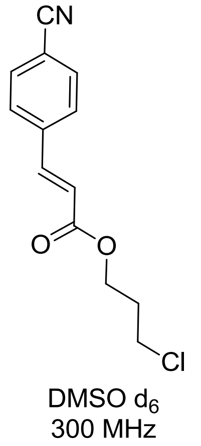

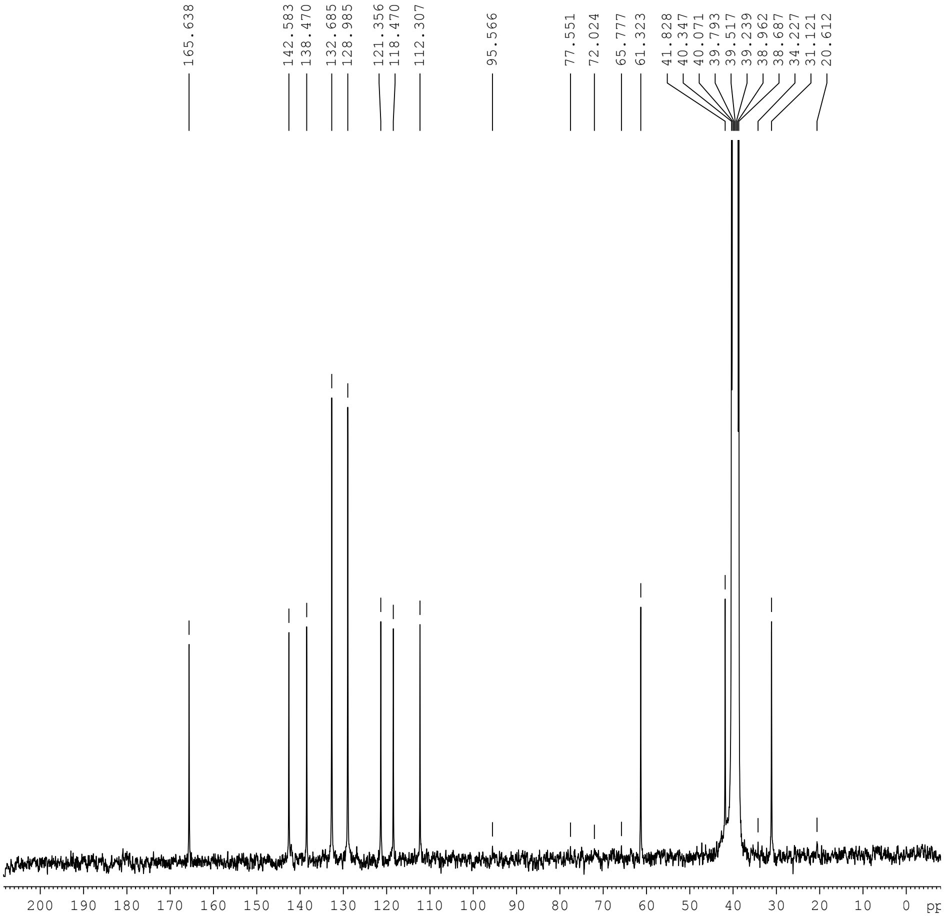

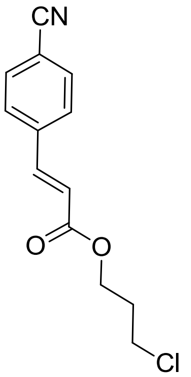

DMSO d_6
75 MHz

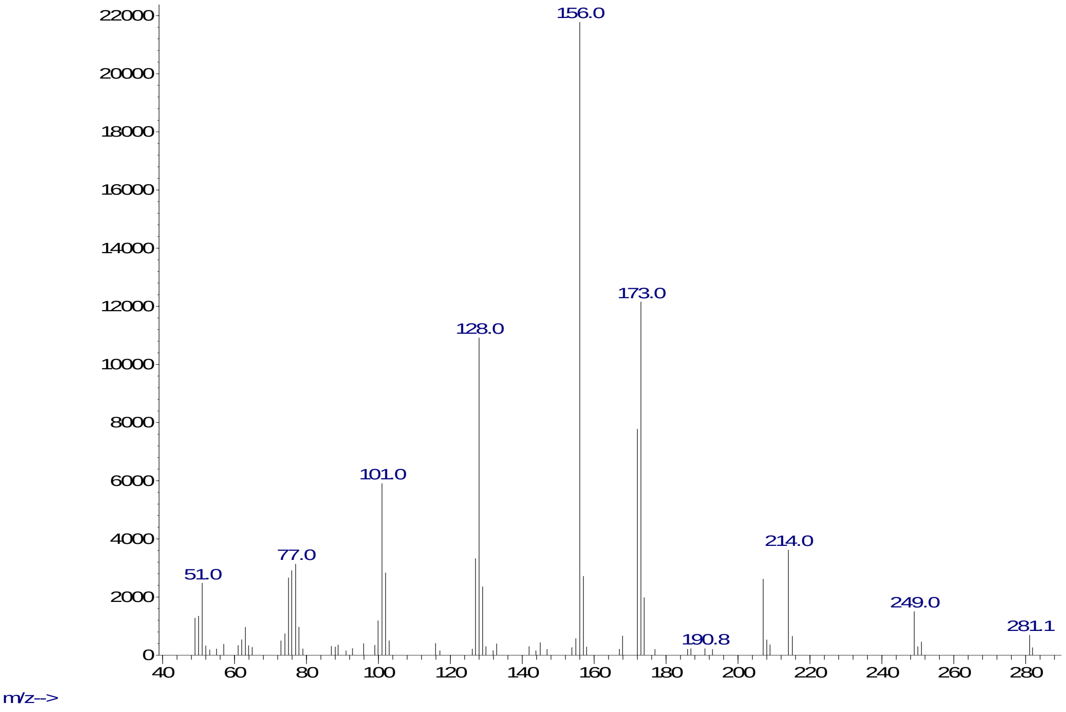

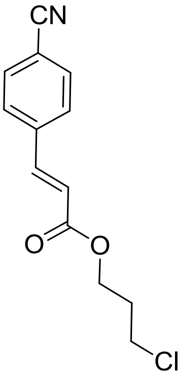

(*E*)-1-Nitro-4-styrylbenzene (**3ca**):

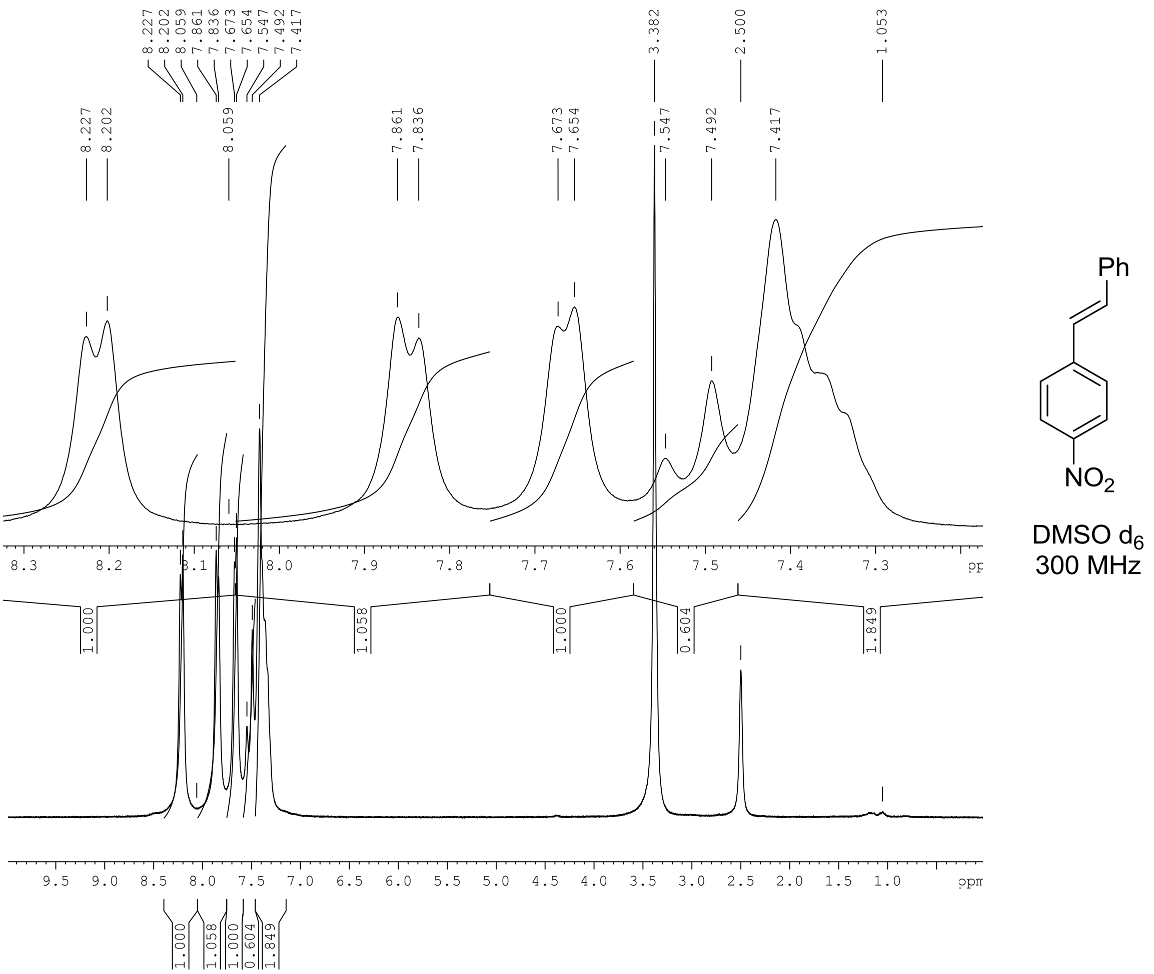

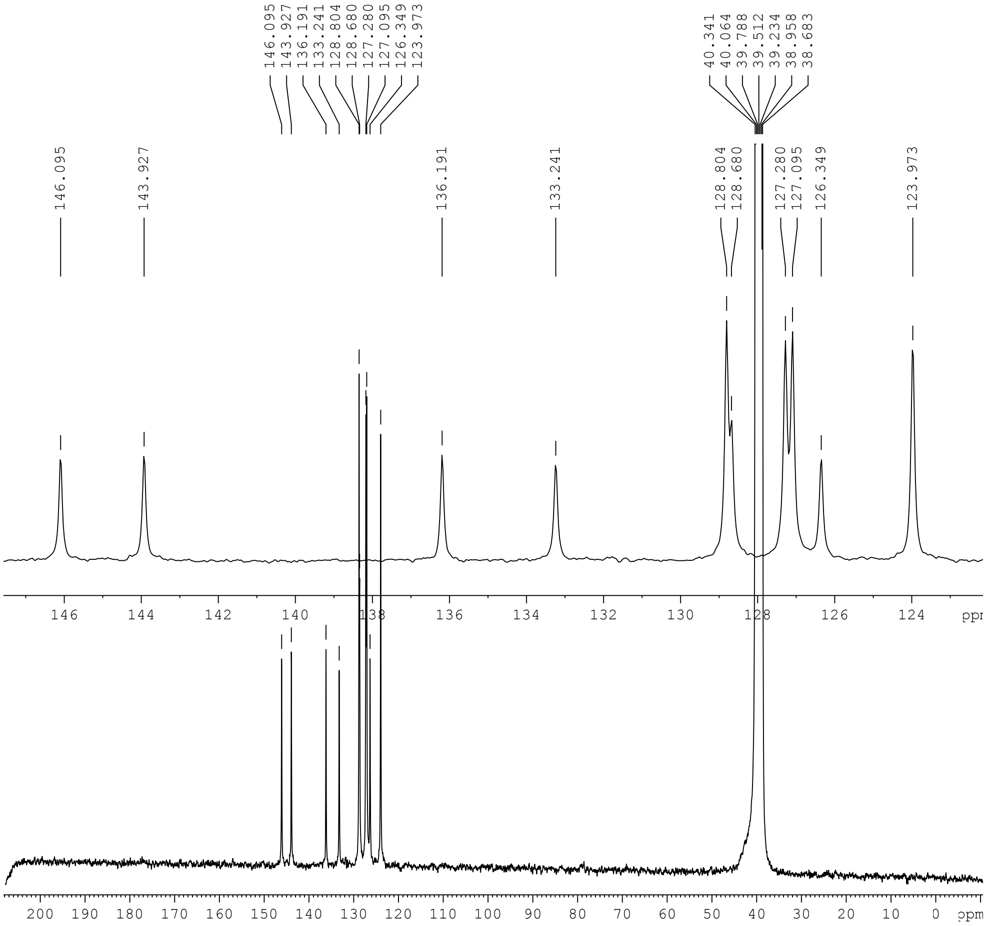

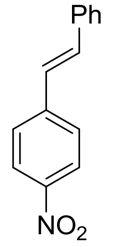

DMSO d_6
75 MHz

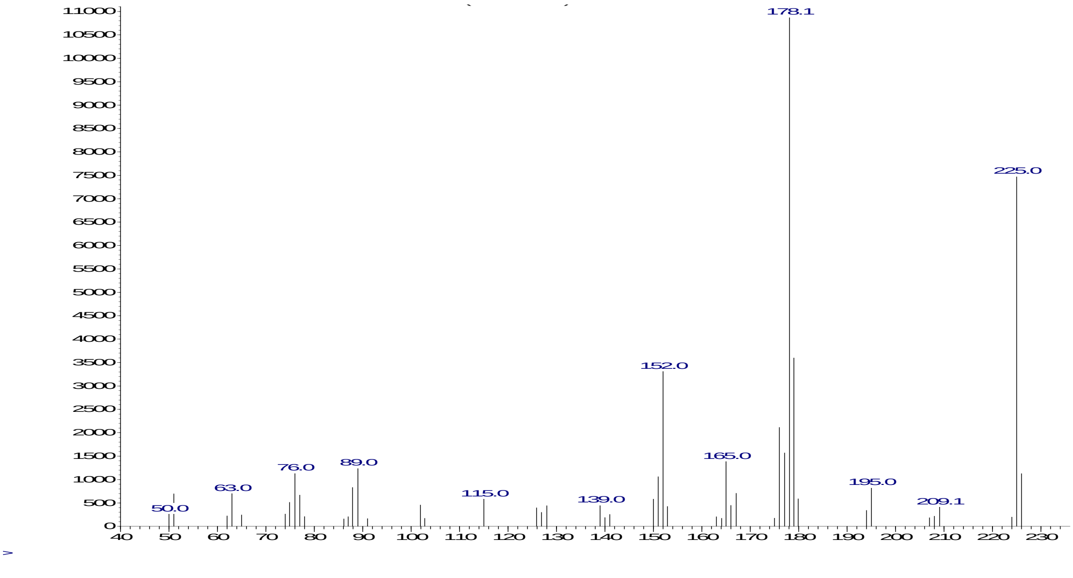

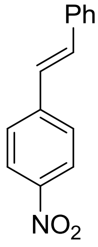

(*E*)-1-Nitro-3-styrylbenzene (**3cb**):

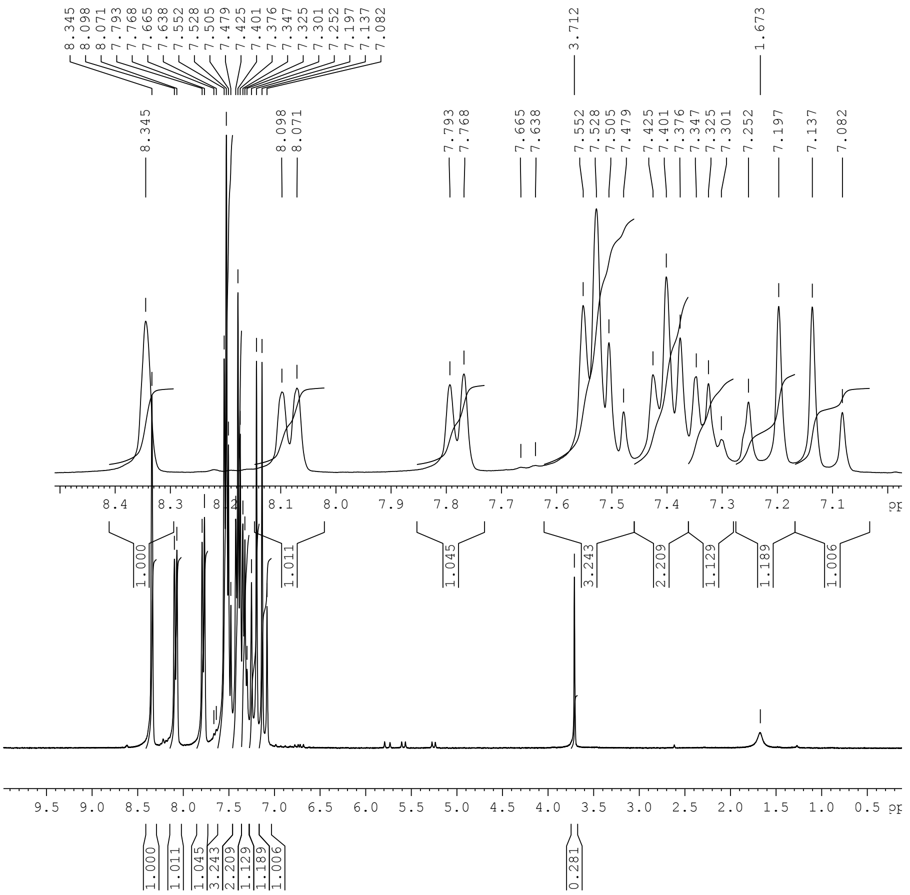

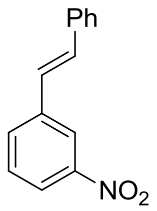

DMSO d_6
300 MHz

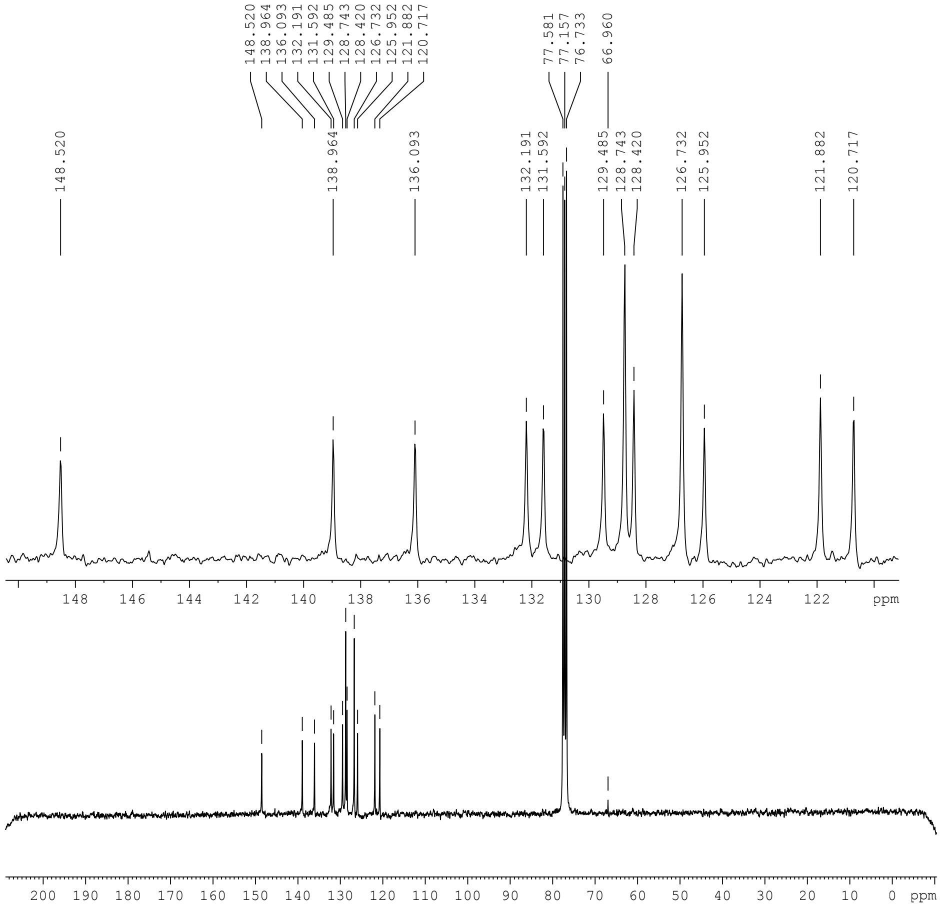

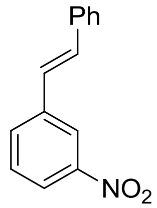

DMSO d_6
75 MHz

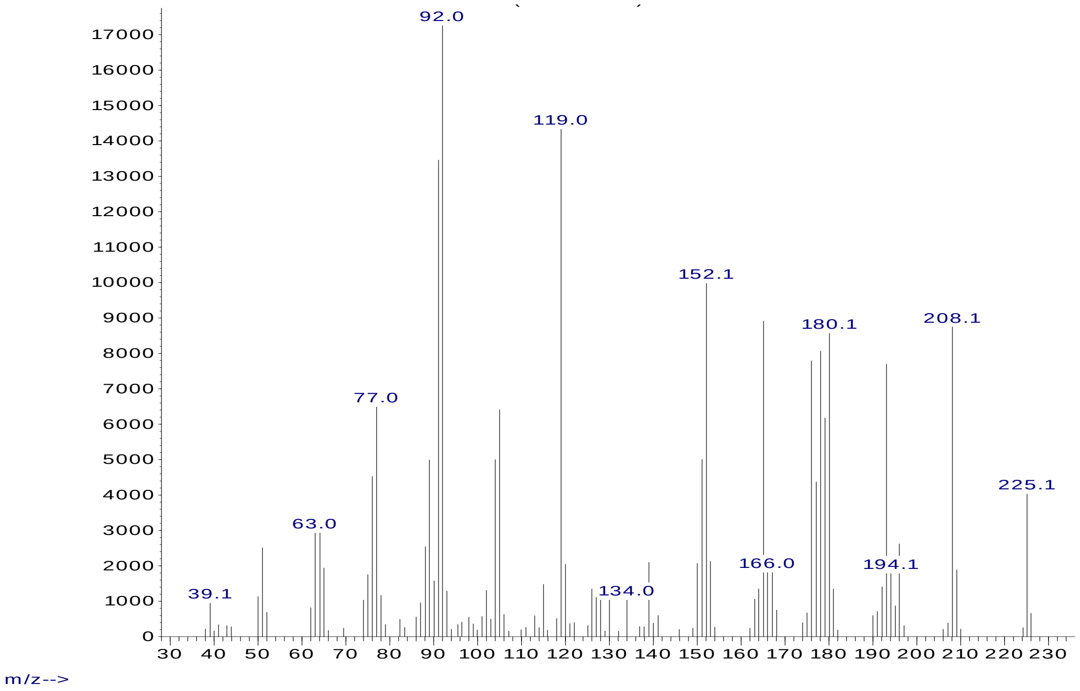

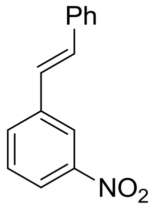

(*E*)-1-methoxy-4-styrylbenzene (**3ce**):

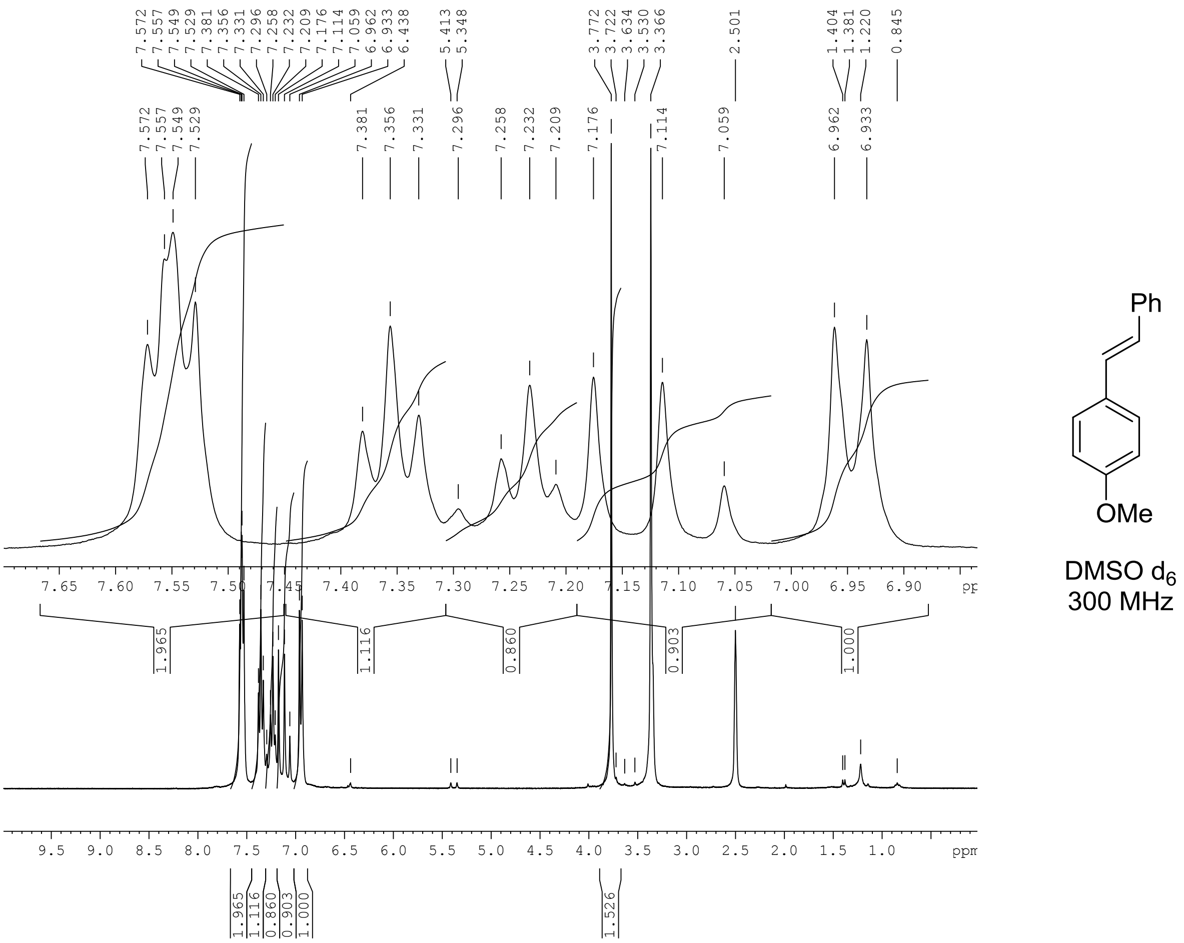

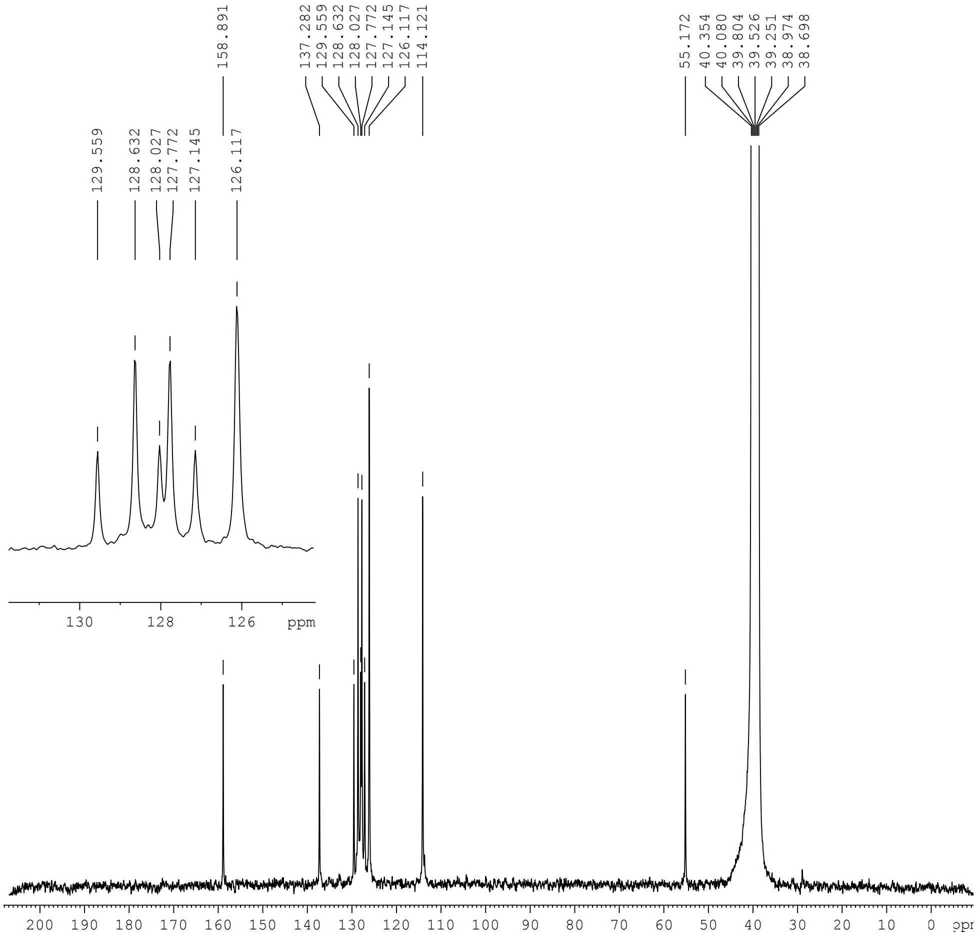

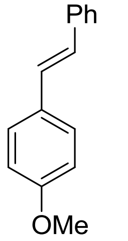

DMSO d_6
75 MHz

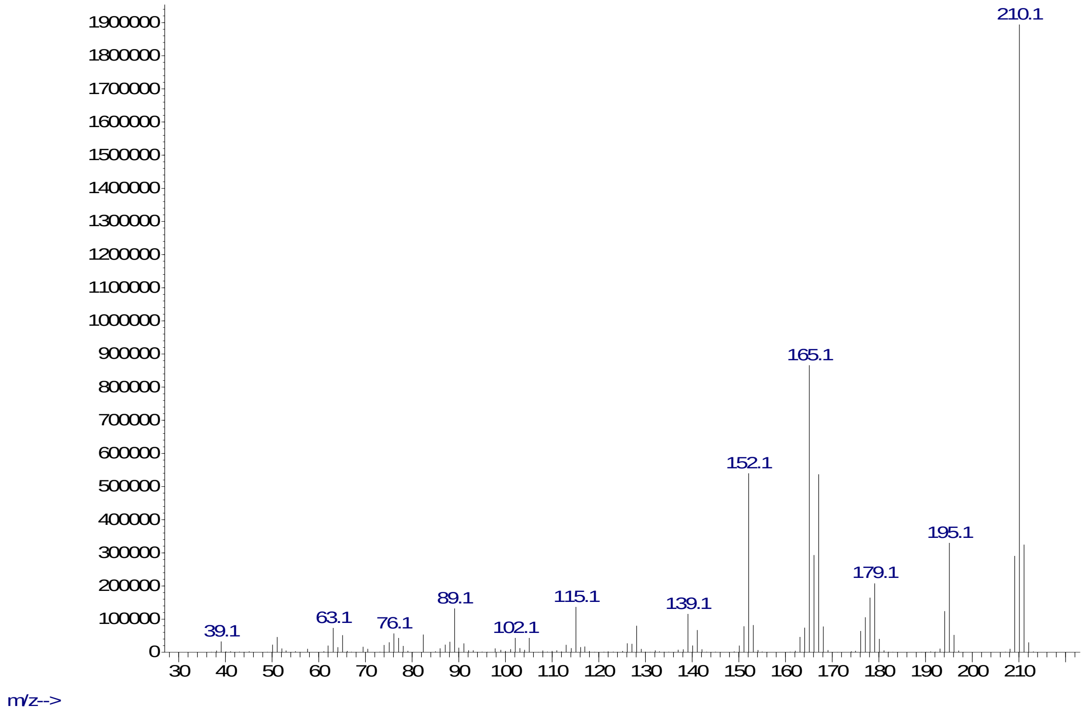

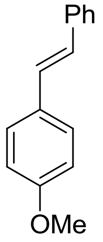

Methyl (*E*)-4-styrylbenzoate (**3cf**):

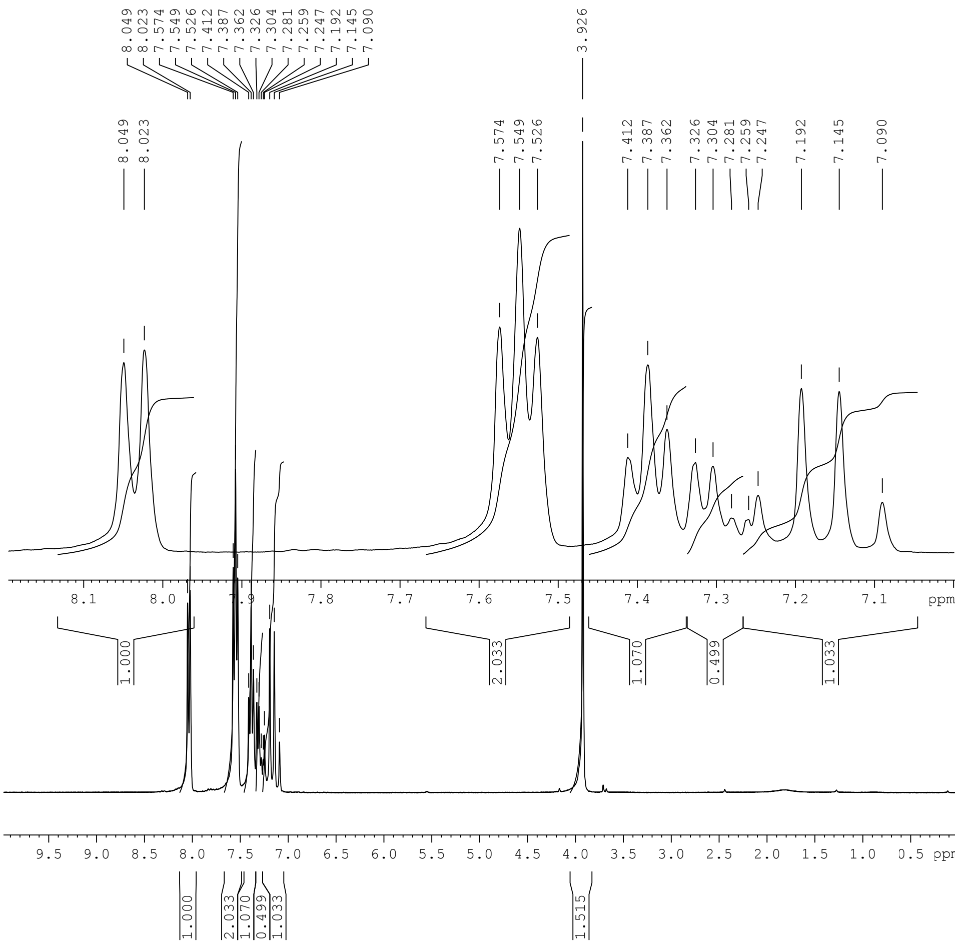

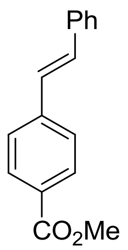

DMSO d_6
300 MHz

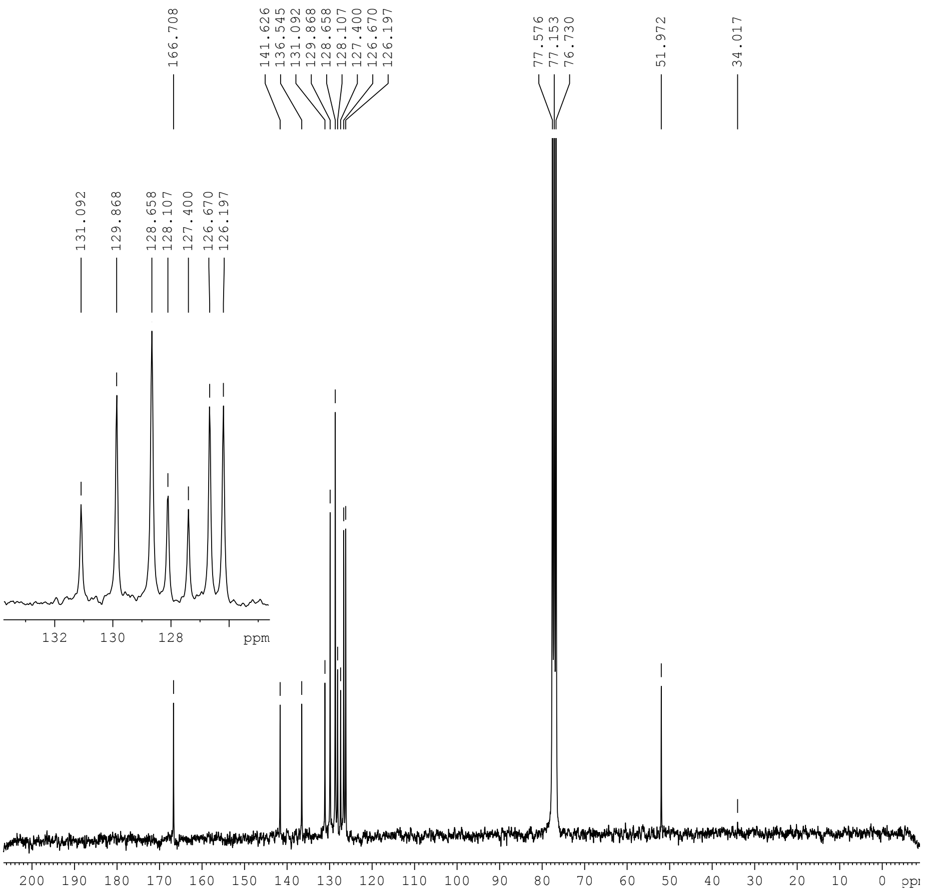

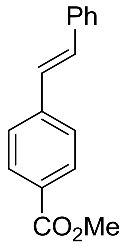

DMSO d_6
75 MHz

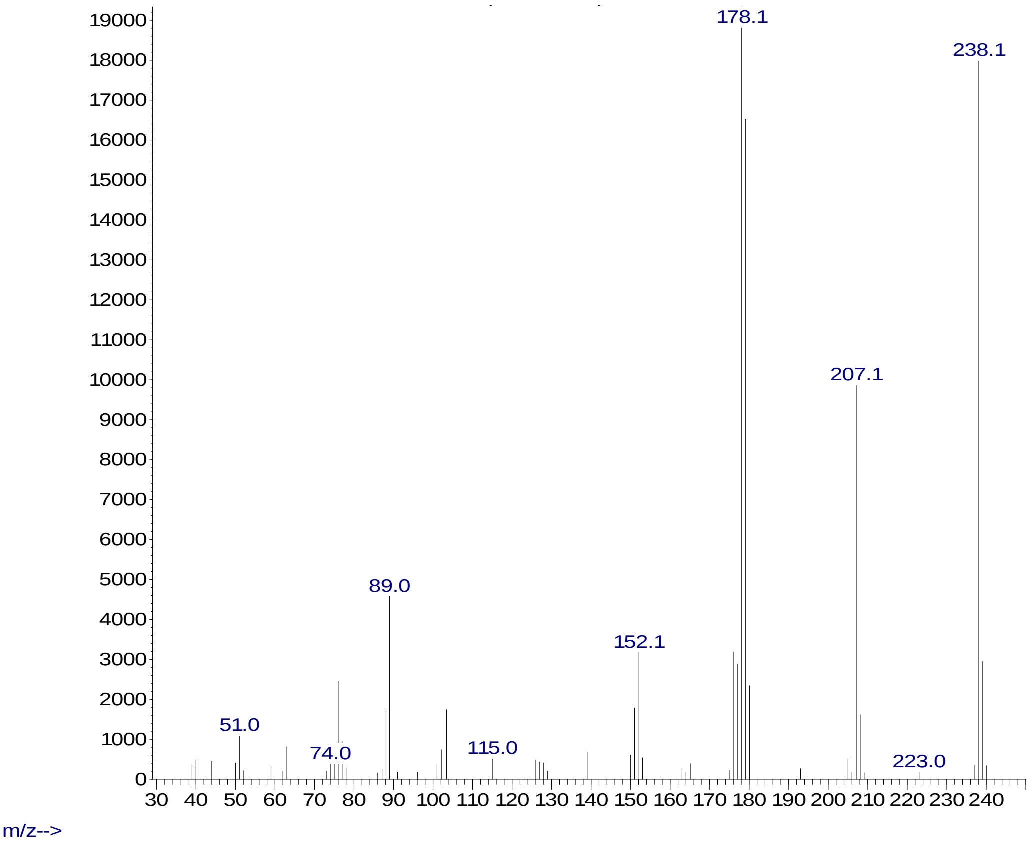

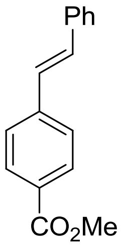

References

1. Filimonov, V. D.; Trusova, M.; Postnikov, P.; Krasnokutskaya, E. a.; Lee, Y. M.; Hwang, H. Y.; Kim, H.; Chi, K. W. *Org. Lett.* **2008**, *10*, 3961-3964.

2. Dai, M.; Liang, B.; Wang, C.; Chen, J.; Yang, Z. *Org. Lett.* **2004**, *6*, 221-224.

3. Dai, M.; Liang, B.; Wang, C.; You, Z.; Xiang, J.; Dong, G.; Chen, J.; Yang, Z. *Adv. Synth. Catal.* **2004**, *346*, 1669-1673.

4. Zhang, Z.; Wang, Z. *J. Org. Chem.* **2006**, *71*, 7485-7487.

5. Andrus, M. B.; Song, C.; Zhang, J. *Org. Lett.* **2002**, *4*, 2079-2082.

6. Modak, A.; Mondal, J.; Sasidharan, M.; Bhaumik, A. *Green Chem.* **2011**, *13*, 1317-1331.

7. Saiyed, A. S.; Patel, K. N.; Kamath, B. V.; Bedekar, A. V. *Tetrahedron Lett.* **2012**, *53*, 4692-4696.
